# Histone demethylase IBM1-mediated meiocyte gene expression ensures meiotic chromosome synapsis and recombination

**DOI:** 10.1371/journal.pgen.1010041

**Published:** 2022-02-22

**Authors:** Chengpeng He, Zhiyu Chen, Yiyong Zhao, Yue Yu, Hongkuan Wang, Cong Wang, Gregory P. Copenhaver, Ji Qi, Yingxiang Wang

**Affiliations:** 1 State Key Laboratory of Genetic Engineering and Ministry of Education Key Laboratory of Biodiversity Science and Ecological Engineering, Institute of Plant Biology, School of Life Sciences, Fudan University, Shanghai, China; 2 Department of Biology and the Integrative Program for Biological and Genome Sciences, University of North Carolina at Chapel Hill, Chapel Hill, North Carolina, United States of America; 3 Lineberger Comprehensive Cancer center, University of North Carolina School of Medicine, Chapel Hill, North Carolina, United States of America; Cornell University, UNITED STATES

## Abstract

Histone methylation and demethylation play important roles in plant growth and development, but the involvement of histone demethylation during meiosis is poorly understood. Here we show that disruption of *Arabidopsis thaliana INCREASE IN BONSAI METHYLATION 1* (*IBM1*) causes incomplete synapsis, chromosome entanglement and reduction of recombination during meiosis, leading to sterility. Interestingly, these *ibm1* meiotic defects are rescued by mutations in either *SUVH4*/*KYP* or *CMT3*. Using transcriptomic analyses we show that mutation of IBM1 down-regulates thousands of genes expressed in meiocytes, and that expression of about 38% of these genes are restored to wild type levels in *ibm1 cmt3* double mutants. Changes in the expression of 437 of these, including the *ARABIDOPSIS MEI2-LIKE AML3-5* genes, are correlated with a significant reduction of gene body CHG methylation. Consistently, the *aml3 aml4 aml5* triple have defects in synapsis and chromosome entanglement similar to *ibm1*. Genetic analysis shows that *aml3 aml4 aml5 ibm1* quadruple mutants resembles the *ibm1* single mutant. Strikingly, over expression of *AML5* in *ibm1* can partially rescue the *ibm1* meiotic defects. Taken together, our results demonstrate that histone demethylase IBM1 is required for meiosis likely via coordinated regulation of meiocyte gene expression during meiosis.

## Author summary

Meiosis is essential for sexual reproduction in most eukaryotes, including plants. In recent years, epigenetic modification such as DNA methylation, histone modifications and histone variants has been shown to play essential roles in meiosis likely by regulating chromatin structure and gene expression. However, the role of histone demethylases in regulating meiotic progression remains largely uncharacterized. Here we show that INCREASE IN BONSAI METHYLATION 1 (IBM1) is required for normal meiotic progression in *Arabidopsis*. Its mutation causes multiple meiotic defects of synapsis and recombination, and reduced fertility. Genetic analyses demonstrate that these *ibm1* defects are restored by mutations in either *SUVH4/KYP* (*SUPPRESSOR OF VARIEGATION HOMOLOG 4/ KRYPTONITE*) or *CMT3* which are associated with DNA CHG methylation. We further show that IBM1 affects expression levels of a large number of genes specifically in meiocytes. Most of them are rescued in *ibm1 cmt3* double mutant and are highly correlated with a significant reduction of gene body CHG methylation, including known meiotic *Arabidopsis-mei2-like genes AML3-5*. Consistently, the *aml3/4/5* triple mutants shows similar meiotic defects to *ibm1* single mutant. Together, our results provide demonstration of IBM1 in meiosis, increasing the spectrum of substrate specificity of key epigenetic regulator histone demethylase.

## Introduction

Meiosis reduces ploidy by half during the production of gametes and is essential for most eukaryotic reproduction [1]. During meiosis, parental chromosomes interact and reciprocally exchange DNA segments in a process known as crossing-over. Crossovers (COs) are required to ensure accurate chromosome segregation and also generate novel allelic combinations in gametes. In flowering plants, meiosis takes place within male anthers and female ovaries which are surrounded by several layers of mitotic cells. Transcriptomic analyses has demonstrated that more than 50% of all protein coding genes are expressed in *Arabidopsis thaliana* [2] and *Zea mays* [3] male meiocytes. Molecular genetic studies have shown that approximately 100 genes have significant roles in *Arabidopsis* meiosis [4,5], but the function of most of these genes remains to be determined. Recently, evidence supporting a meiotic role for epigenetic modifications such as DNA methylation, histone modifications and histone variants has emerged [5,6], but the underlying molecular mechanisms remain largely unclear.

In plants, DNA methylation at cytosines is observed in three primary contexts: CG, CHG and CHH (H = A, T or C) [7]. Mutant analysis in *Arabidopsis* has shown that altering CG, CHG or CHH methylation redistributes COs without changing the total number [8–11]. In comparison to DNA methylation, histone methylation seems to be more important for meiotic progression. In mammals and yeast, PRDM9/Spp1 controls meiotic recombination hotspots by catalyzing H3K4 trimethylation (H3K4me3) [12–14]. Although plants lack a PRDM9 homolog, their CO hotspots are also correlated with H3K4me3 as well as the histone variant H2A.Z [15]. These results suggest that the positive association of CO hotspots with H3K4me3 is conserved in yeasts [16], mammals [17] and plants [15,18]. The potential roles of other histone tail modifications in meiosis are less well understood. In mice, the H3K9me1/2 methyltransferase G9a is required for synapsis and double strand break (DSB) repair [19]. In plants H3K9me2 is catalyzed by SUVH4/KYP (SUPPRESSOR OF VARIEGATION HOMOLOG 4/KRYPTONITE), SUVH5 and SUVH6 and is correlated with CHG methylation through a regulatory loop [20–23]. However, the *suvh4 suvh5 suvh6* triple mutants have normal fertility and meiotic progression [11,19] with increased CO frequencies in pericentromeric regions [11]. Given that non-CG methylation and H3K9me2 are enriched at highly condensed heterochromatic regions in *Arabidopsis* [11,18], H3K9me2 has been proposed to modulate meiotic recombination by altering chromatin structure.

Whether meiotic progression requires the removal of various methylated histones is an open question. We found previously that the *Arabidopsis* H3K4 demethylase JMJ16 participates in meiotic chromosome condensation, and that *jmj16* mutants exhibit slight reductions in fertility but relatively normal meiotic progression [24]. Transcriptomic analyses of purified meiocytes showed that nearly all histone demethylases are active [2], suggesting a role in meiosis. Moreover, meiotically expressed genes are negatively correlated with gene body CHG methylation [25]. In *Arabidopsis* somatic cells, H3K9me2 can be demethylated by IBM1 (INCREASE IN BONSAI METHYLATION 1) [26–28]. Mutation of *IBM1* induces elevation of CHG methylation within gene body or gene-like TEs (transposable elements) accompanied by reduced gene expression, but not in repetitive sequences or TEs [29–31]. However, it is not clear whether H3K9me2/CHG demethylation is also required for meiosis.

IBM1 belongs to the conserved KDM3 (LYSINE-SPECIFIC DEMETHYLASE 3) family of demethylases and has putative H3K9me2 demethylase activity [32,33]. *Arabidopsis* has six members: KDM3A-KDM3F [32]. KDM3A/JMJ24 does not have H3K9me2 demethylase activity because they lack a conserved cofactor binding sites [34,35], while KDM3C/JMJ25/IBM1, KDM3D/JMJ27 and KDM3F/JMJ29 can demethylate H3K9me2 [36,37]. However, only *ibm1* mutants have reduced fertility, suggesting a role in meiosis. Here we show that *Arabidopsis IBM1* is required for proper chromosome synapsis and recombination during meiosis. We further demonstrate that *ibm1* mutant phenotypes can be rescued by mutations in either *CMT3* or *SUVH4*/*KYP*. We use transcriptional profiling to show that IBM1 regulates thousands of meiotically expressed genes, and that there is a correlation between genes that exhibit the restoration of normal expression levels in *ibm1 cmt3* mutants and gene body CHG methylation, including genes in the *AML* (*ARABIDOPSIS MEI2 LIKE*) family. Consistent with these findings, the *aml3 aml4 aml5* triple mutants exhibit incomplete synapsis and chromosome entanglement similar to *ibm1* single mutant. Genetic analysis supports the idea that AML genes likely acts in the same pathway as IBM1. Strikingly, over expression of *AML* genes in *ibm1* can partially rescue the *ibm1* phenotypes. In summary, we demonstrate that histone H3K9 demethylase IBM1 is required for meiosis in plants, significantly broadening our mechanistic understanding of the role of histone demethylation in meiosis.

## Results

### *IBM1* is required for meiosis in *Arabidopsis*

**Fig 1:**
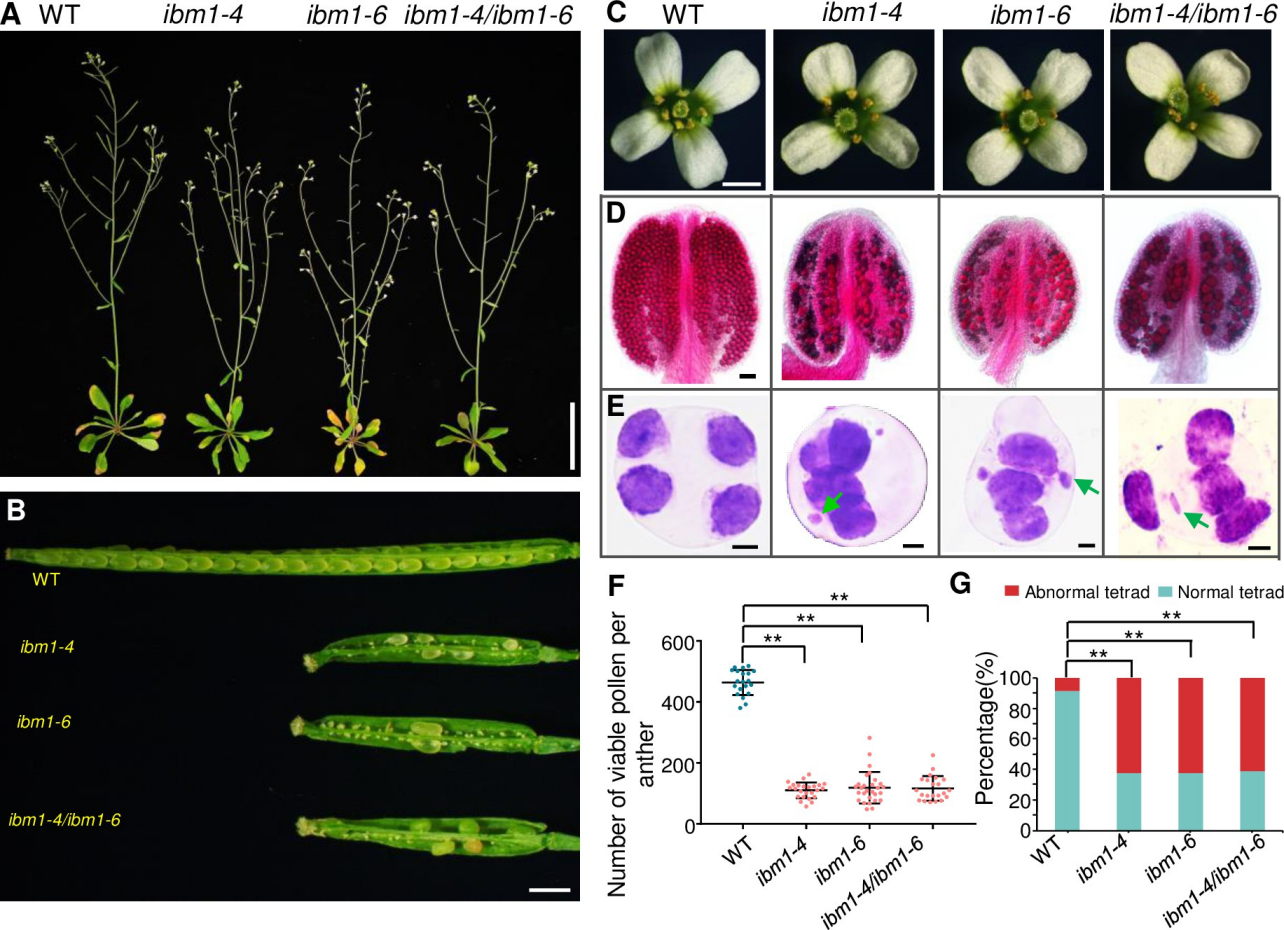
IBM1 is required for male fertility. (A) Plant images of wild type (WT), *ibm1-4*, *ibm1-6* and *ibm1-4*/*ibm1-6* whole plants. (B) Siliques from WT, *ibm1-4*, *ibm1-6*, and *ibm1-4*/*ibm1-6*. (C)—(E) Flowers (C), anthers after Alexander staining (D) and Toluidine blue stained tetrad-stage meiocytes (E) from WT (n = 112), *ibm1-4* (n = 228), *ibm1-6* (n = 332), and *ibm1-4*/*ibm1-6* (n = 248) plants. Green arrows in (E) indicate micronuclei. (F) Quantification of viable pollen grains per anther from WT, *ibm1-4*, *ibm1-6* and *ibm1-4*/*ibm1-6* plants. ** represents p-value< 0.01 in two-tailed student *t* test. The data are shown as mean ± SD. (G) Histogram of different types of tetrads. ** represents p-value< 0.01 in two-tailed chi-square test. Scale bar: (A): 5 cm, (B): 1 mm, (C): 1 mm, (D): 50 μm, (E): 5 μm.

Our meiocyte transcriptome shows that most of histone demethylases are preferentially expressed in meiocytes [2], but none of them plays main function in fertility except for IBM1 [26]. To test the role of IBM1 in meiosis, we obtained two T-DNA insertion lines, *ibm1-4* (SALK_035608, insertion in exon 8) [26,27] and phenotypic uncharacterized *ibm1-6* (SALK_006042, insertion in exon 6) (S1A Fig). We used quantitative RT-PCR (RT-qPCR) to demonstrate that no *IBM1* transcript spanning the insertion site was detected in *ibm1-6*, indicating a potential null allele (S1B Fig). Homozygous *ibm1-4* and *ibm1-6* plants grew slightly slower compared with WT, but showed dramatically reduced fertility with shorter siliques and fewer seeds (Fig 1A and 1B and S1 Table), consistent with previous findings [26,28]. WT and *ibm1* have indistinguishable floral morphology, but the mutant has fewer viable pollens on the stigma suggesting a defect in male meiosis (Fig 1C). To examine whether female fertility is compromised, we performed reciprocal crosses between WT and *ibm1* homozygous plants and found that both male and female fertility are defective (S1 Table) with male fertility more severely affected. Alexander staining shows that WT anthers have 464 ± 41 (n = 20) viable pollen grains, while *ibm1-4* and *ibm1-6* anthers have fewer with 110 ± 26 (n = 24) and 119 ± 52 (n = 28) respectively (Fig 1D and 1F). Toluidine blue staining of tetrad-stage meiocytes reveals four uniform microspores in WT, but the presence of micronuclei in *ibm1-4* and *ibm1-6* (Fig 1E and 1G) implies a defect in meiosis. To validate that the fertility defects we observed are due solely to lesions in *IBM1*, we generated *ibm1-4*^-/+^/*ibm1-6*^*-*/+^ compound heterozygous plants, which also have short siliques, inviable pollen grains and abnormal tetrad-stage meiocytes (Fig 1). In addition, we used trans-complementation with the WT *IBM1* coding sequence, with a C-terminal in-frame 3×FLAG tag fusion, expressed by the native *IBM1* promoter to confirm that the phenotypes we observed are caused by mutations in the *IBM1* locus (S1C Fig). The *pIBM1*::*IBM1-FLAG* transgene is able to rescue the fertility of *ibm1-6*^*-*/-^ homozygous plants and restore normal levels of *IBM1* expression (S1D–S1F Fig), confirming that IBM1 is required for normal fertility and meiosis in *Arabidopsis*.

### *IBM1* mutations cause chromosome synapsis and meiotic recombination defects

**Fig 2:**
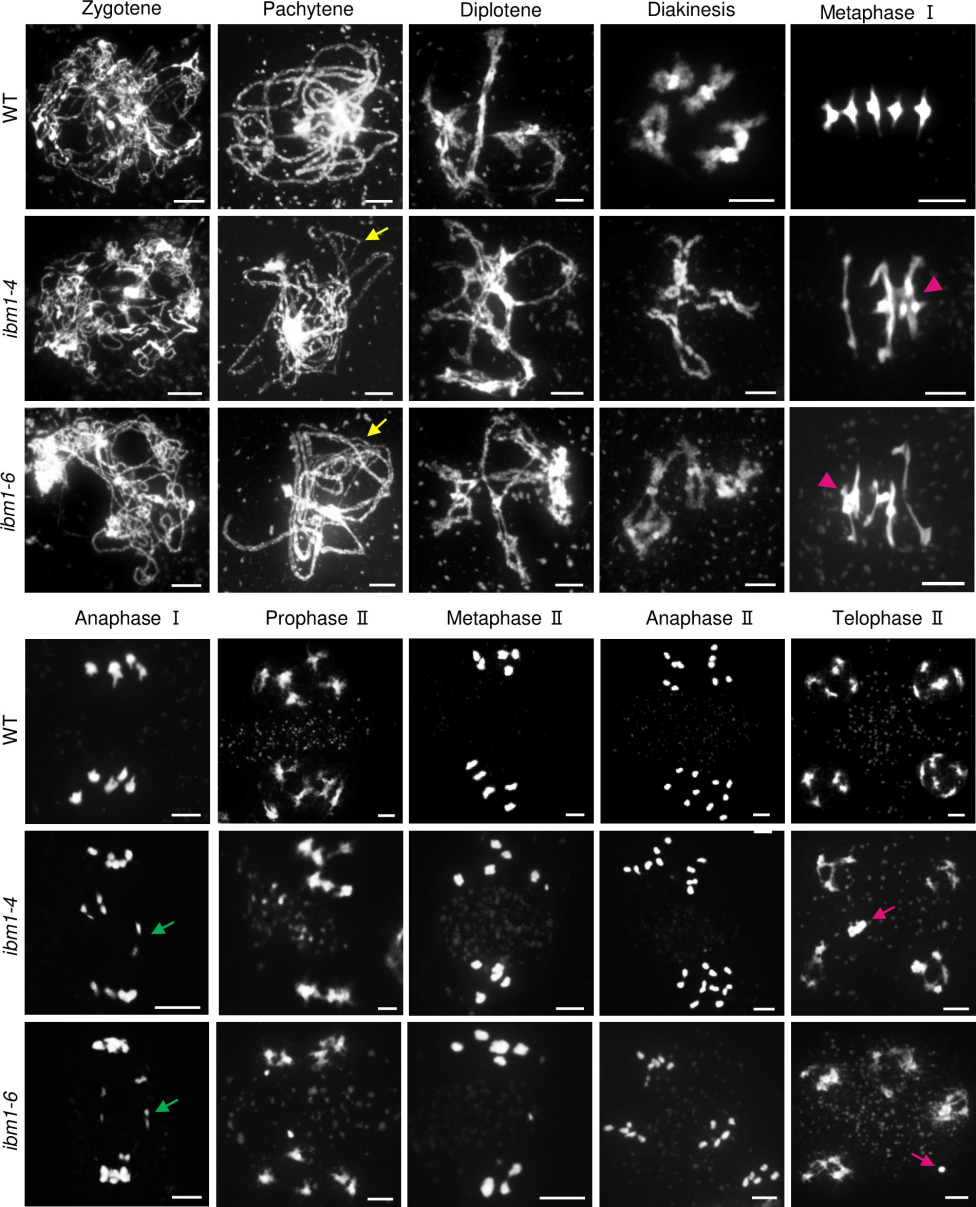
DAPI stained chromosome spreads from WT and *ibm1* mutants. Chromosome spreads showing meiotic chromosome morphologies from WT, *ibm1-4* and *ibm1-6* meiocytes. Yellow arrows on mutant pachytene chromosomes indicate the unsynapsed regions. Magenta triangles on metaphase I chromosomes show chromosome entanglements. Green arrows on anaphase I mutant chromosomes indicate chromosome fragments or lagging chromosomes. The magenta arrows on telophase II show chromosome fragments. Scale bar: 5 μm.

We stained WT and *ibm1* chromosomes spreads with 4’,6-diamidino-2-phenylindole (DAPI) to examine potential defects in meiotic chromosome morphology. The chromosome configurations of *ibm1-4*, *ibm1-6* and WT are indistinguishable prior to pachytene (Fig 2). At pachytene, WT chromosomes are fully synapsed, while 51.8% of *ibm1-4* (n = 83) and 54.1% of *ibm1-6* (n = 109) meiocytes have incompletely synapsed chromosomes (Figs 2 and S2A). By metaphase I the chromosomes in WT meiocytes are organized into five bivalents, but the *ibm1* chromosomes that form bivalents have an elongated morphology compared to WT (Fig 2). The compact bivalent morphology seen in WT is consistent with at least 2 COs on each chromosome, while the elongated bivalent morphology is indicative of a single CO suggesting a reduction of chiasmata in *ibm1*. Moreover, 79.7% (n = 74) and 73.7% (n = 103) of the *ibm1-4* and *ibm1-6* meiocytes had entangled chromosomes (Figs 2 and S2C), which suggest non-homologous associations. We used fluorescence in situ hybridization (FISH) assays with a 5S rDNA probe to confirm that the chromosome entanglements involve nonhomologous chromosome interactions in *ibm1-4* (9/18 metaphase I cells) and *ibm1-6* (6/11 metaphase I cells) (S3 Fig). In subsequent stages chromosome fragmentation and unequal segregation were observed in *ibm1* but not WT meiocytes (Figs 1 and S2). In addition, consistent with the observation of micronuclei in tetrad-stage microspores in *ibm1*, chromosome fragmentation was observed in *ibm1* telophase II meiocytes. These results are further confirmed by using FISH with a centromeric probe (S2D Fig).

### Synapsis is compromised in *ibm1*

**Fig 3:**
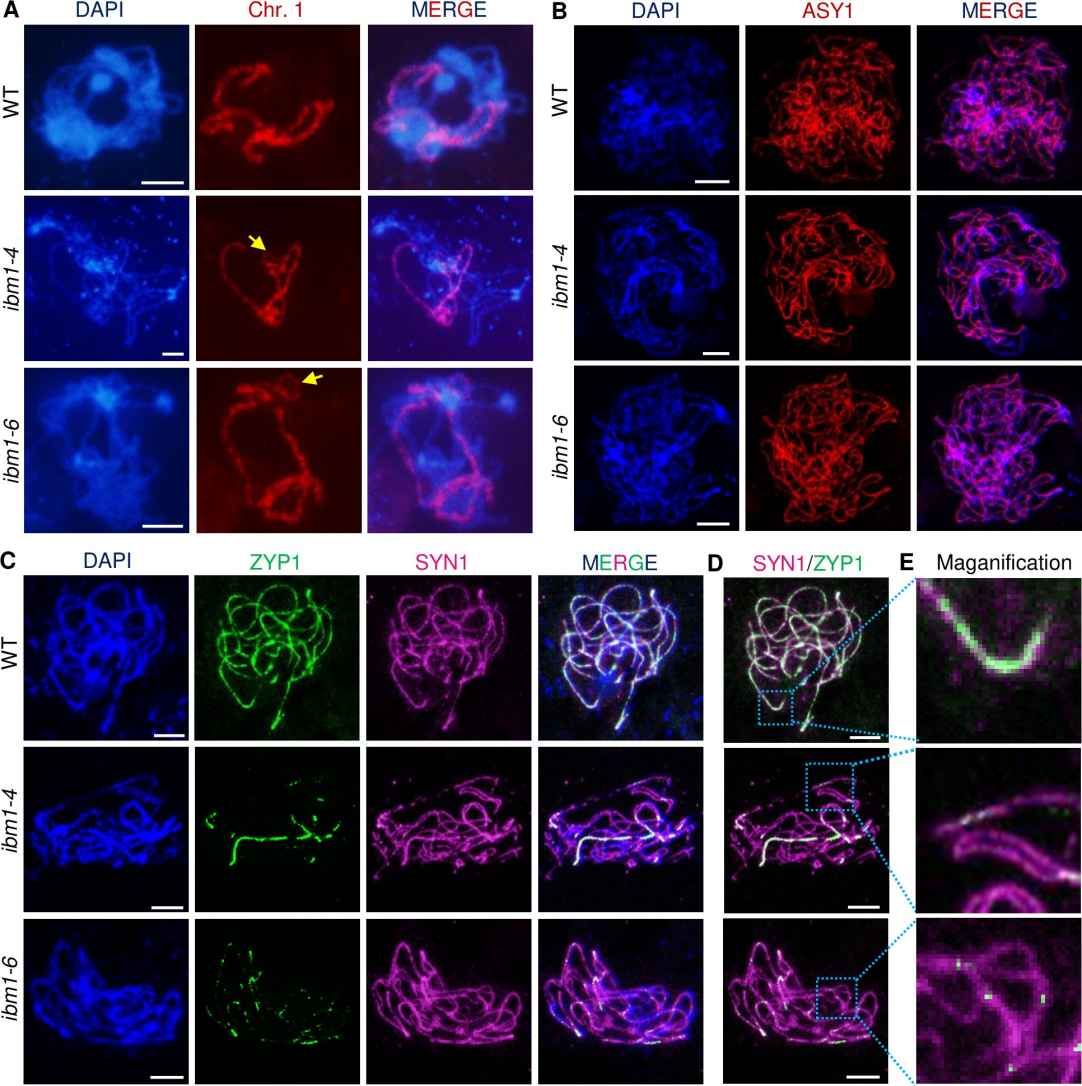
IBM1 is required for synapsis. (A) Image showing dual signals of DAPI (blue) with chromosome 1 painting (red) at pachytene in WT, *ibm1-4* and *ibm1-6*. (B) Immunofluorescence of ASY1 (red) in WT, *ibm1-4* and *ibm1-6* mutants at zygotene. (C) Localization of ZYP1 (green) and SYN1 (magenta) with DAPI (blue) at pachytene in WT, *ibm1-4* and *ibm1-6* meiocytes. (D) The merged ZYP1 (green) and SYN1 (magenta) signals from (C). The blue dotted-lined rectangles are magnified in (E) and show the regions with incomplete synapsis. Scale bar: 5 μm.

To further characterize the incomplete synapsis in *ibm1*, we performed chromosome 1 painting using oligonucleotide probes labeling euchromatins. In pachytene meiocytes, two homologous chromosome 1 in WT are fully paired and formed a thread-like signal. In contrast, the *ibm1* mutants have unsynapsed regions (Fig 3A). We further conducted immunofluorescence assays using antibodies against the lateral element ASY1 [38] and transverse filament ZYP1 [39] components of the synaptonemal complex (SC). At zygotene, no obvious differences in the distribution of ASY1 and ZYP1 signals were observed in WT or *ibm1* meiocytes (Figs 3B and S4A). At pachytene, when homologous chromosomes are fully synapsed, ASY1 is gradually removed from the chromosomes and WT and *ibm1* meiocytes remain indistinguishable (S4B Fig), indicating normal assembly of the lateral element in *ibm1*. In contrast, ZYP1 has a continuous linear distribution pattern in WT pachytene meiocytes (n = 31), but is discontinuously distributed in 45.4% *ibm1-4* (n = 44) and 41.1% *ibm1-6* (n = 51) meiocytes (Fig 3C–3E), suggesting that the incomplete synapsis in *ibm1* involves dysregulation of the transverse filament component of the SC.

### IBM1 is dispensable for DSB formation but required for repair

**Fig 4:**
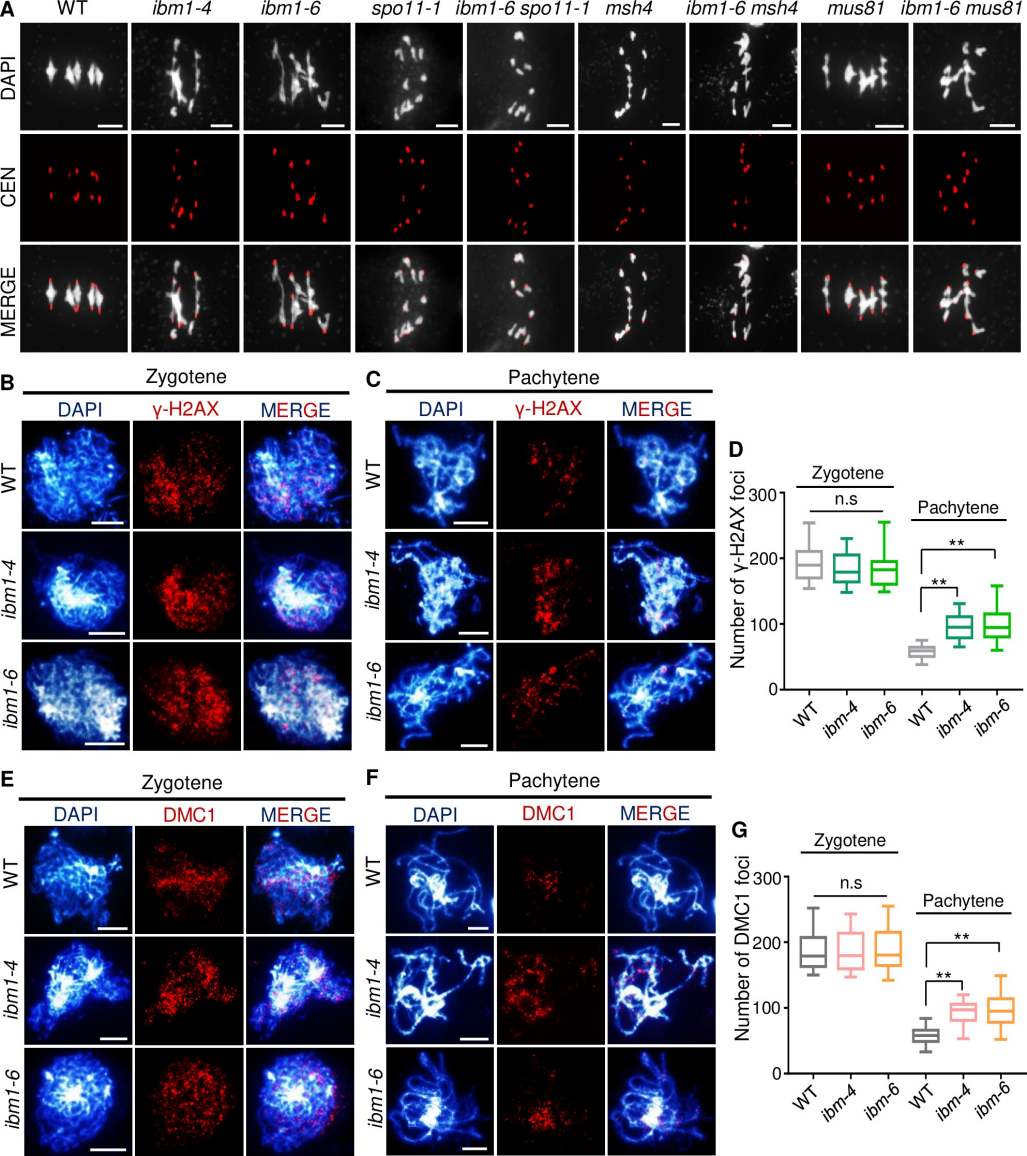
IBM1 is required for meiotic recombination. (A) Images showing FISH with centromere probe of WT, *ibm1-4*, *ibm1-6*, *spo11-1*, *ibm1-6 spo11-1*, *msh4*, *ibm1-6 msh4*, *mus81*, *ibm1-6 mus81* metaphase I chromosome spreads. (B) and (C) Zygotene and pachytene localization of γ-H2AX in WT, *ibm1-4* and *ibm1-6* meiocytes. (D) Quantification of γ-H2AX foci at zygotene and pachytene in WT, *ibm1-4* and *ibm1-6* mutants. (E) and (F) showing zygotene and pachytene localization of γ-H2AX and DMC1 in WT, *ibm1-4* and *ibm1-6* meiocytes. (G) Quantification of DMC1 at zygotene and pachytene in WT, *ibm1-4* and *ibm1-6* mutants. Box-plots show the median and interquartile ranges and the whiskers extend to the maximum and minimum of number of foci. ** p-value<0.01 with two-tailed student *t* test. Scale bar: 5 μm.

The occurrence of entangled chromosomes in *ibm1* suggests a defect in meiotic recombination. In *Arabidopsis*, meiotic recombination is initiated by the formation of DSBs catalyzed by SPO11-1 [40] during the leptotene—zygotene transition. To test whether the meiotic defects observed in *ibm1* are dependent on SPO11-1, we crossed *spo11-1* with *ibm1-6* to compare the double mutant with each single mutant. We observed a complete absence of chromosome pairing and no chromosome entanglement in *spo11-1* metaphase I meiocytes and an indistinguishable phenotype in *spo11-1 ibm1-6* double mutant meiocytes (Fig 4A), suggesting that IBM1 acts downstream of SPO11-1.

To investigate how SPO11 mediated DSBs are processed in *ibm1*, we performed immunofluorescence assays using antibodies against the phosphorylated histone H2AX (γ-H2AX) as a marker for DSBs [41]. We observed no significant difference in the number of γ-H2AX foci in WT (194 ± 10; n = 20) and *ibm1-4* (183 ± 25; n = 23; p-value = 0.18) and *ibm1-6* (183 ± 25; n = 22; p-value = 0.17) zygotene meiocytes (Fig 4A and 4C). The number of γ-H2AX foci decreases in WT meiocytes at pachytene (58 ± 10; n = 21). There is also a decrease in γ-H2AX foci in *ibm1-4* (96 ± 20; n = 27) and *ibm1-6* (98 ± 24; n = 22) from zygotene to pachytene, but it is a smaller decrease than that observed in WT (WT vs. *ibm1-4*: p-value = 5.1E-11; WT vs. *ibm1-6*: p-value = 1.0E-7) (Fig 4B and 4C) suggesting a delay in DSB repair. We also used immunofluorescence assays with antibodies against DMC1 (DISRUPTED MEIOTIC cDNA 1), which facilitates single end invasion following DSB formation [42]. We observed no significant difference in the number of DMC1 foci between WT and *ibm1* zygotene meiocytes (Fig 4D and 4F). Similar to the pattern observed with γ-H2AX, the number of DMC1 foci decreased from zygotene WT (187 ± 29; n = 20), *ibm1-4* (185 ± 28; n = 20) and *ibm1-6* (188 ± 31; n = 22) to pachytene WT (58 ± 12; n = 28), *ibm1-4* (94 ± 15; n = 32) and *ibm1-6* (97 ± 24; n = 27), but *ibm1* had a relatively smaller decrease (WT vs. *ibm1-4*: p-value = 8.0E-14; WT vs. *ibm1-6*: p-value = 4.1E-9) (Fig 4E and 4F). These results support the idea that IBM1 is not necessary for DSB formation, but is required for subsequent repair processes, which is consistent with the observed chromosome fragmentation phenotype in *ibm1* (Fig 2).

### IBM1 is required for both meiotic type I and type II CO formation

**Fig 5:**
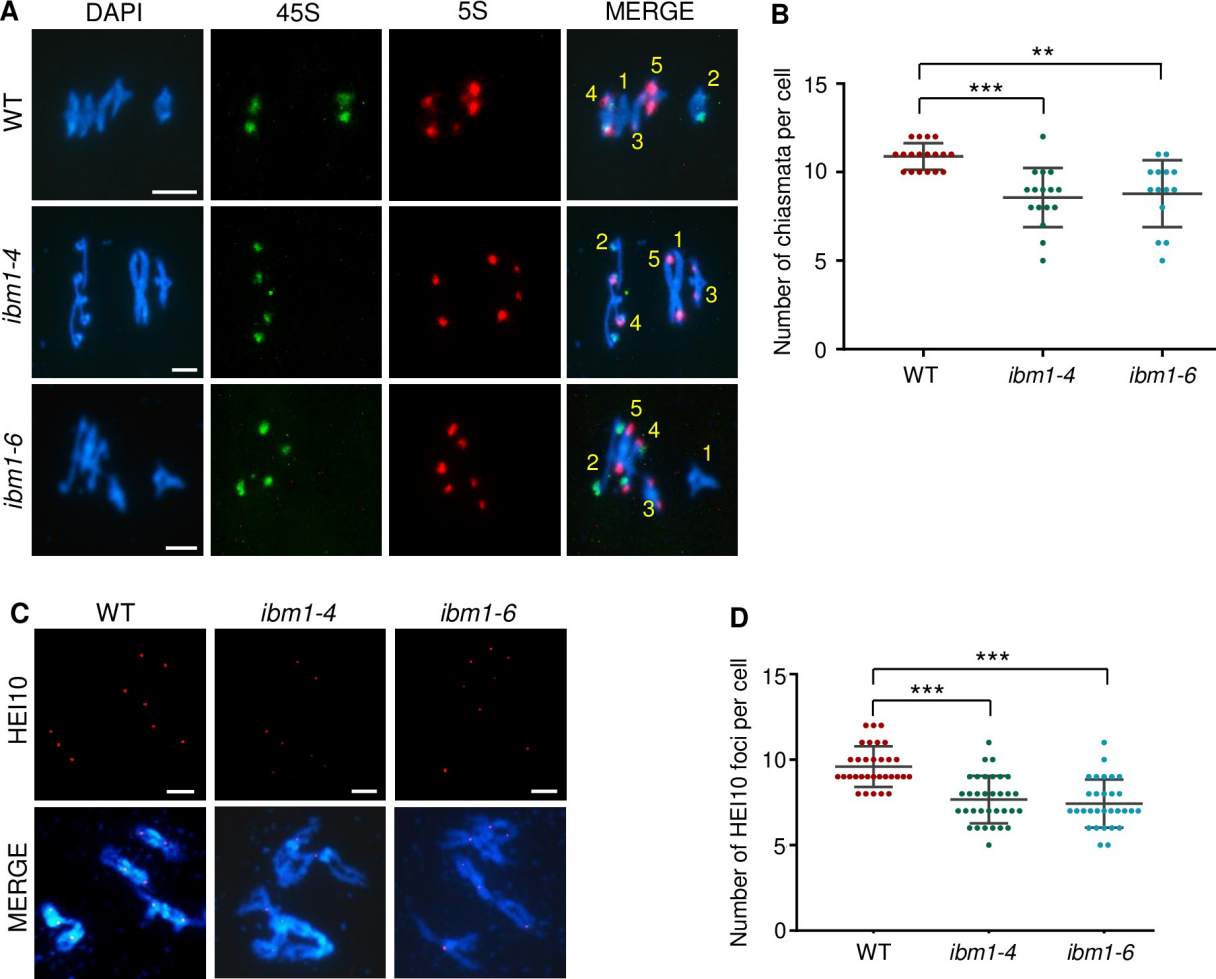
Reduction of COs observed in *ibm1*. (A) 45S (green) and 5S (red) FISH of metaphase I from WT, *ibm1-4*, *ibm1-6*. (B) Quantification of the number of chiasmata in each WT, *ibm1-4* and *ibm1-6* meiocyte. The data are shown as mean ± SD. (C) Immunofluorescence analysis of HEI10 in WT, *ibm1-4*, *ibm1-6* diakinesis chromosome spreads. (D) Quantification of the number of HEI10 foci in each WT, *ibm1-4* and *ibm1-6* meiocyte. The data are shown as mean ± SD. In image (B) and (D), ** represents p-value<0.01, *** represents p-value<0.001 with two-tailed student *t* test. Scale bar: 5 μm.

The occurrence of elongated bivalent in *ibm1* (Fig 2) is indicative of reduction of crossovers (COs), we labeled chromosome with 45S and 5S rDNA probes and quantified the number of COs according to metaphase I chromosome morphology (Fig 5B). In WT, one meiocyte had 10.89 ± 0.76 (n = 18), but the number of CO in *ibm1-4* and *ibm1-6* decreased to 8.56 ± 1.67 (n = 16) and 8.79 ± 1.89 (n = 14) (Fig 5C and S2 Table).

To differentiate the effect of *ibm1* on Type I and II COs, we crossed *ibm1-6* with *msh4* [43] and *mus81* [44]. *Arabidopsis* MSH4 belongs to the ZMM (MSH4, MSH5, MER3, SHOC1, PTD, HEI10, ZIP4) group of proteins that mediate the Type I CO pathway and accounts for 70~85% of the total COs [5]. We observed only 1.3 ± 0.5 bivalents (n = 29) in *msh4* metaphase I meiocytes, while the *ibm1-6 msh4* meiocytes had significantly fewer (0.3 ± 0.6; n = 15; p-value = 6.3E-7), suggesting that the residual COs in *ibm1* are at least partially type I-independent (Fig 5A). *Arabidopsis* MUS81 is an endonuclease that mediates the Type II pathway which produces 5~15% of the total COs [44]. The *mus81* single mutant forms five bivalents at metaphase I (n = 21) (Fig 5A), while the *ibm1-6 mus81* double mutant has significantly fewer (2.8 ± 1.2; n = 25, p-value = 2.1E-9) as does the *ibm1-6* single mutant with countable bivalents (2.9 ± 1.3; n = 38, p-value = 6.3E-12) (Fig 5A). Together, these results indicate that IBM1 influences both Type I and Type II COs.

We further performed immunofluorescence to count HEI10 (HOMO SAPIENS ENHANCER OF INVASION 10) foci at diakinesis, a Type I CO marker [45]. WT meiocytes had 9.6 ± 1.2 (n = 32) HEI10 foci, while *ibm1-4* (7.7 ± 1.4; n = 33; p-value = 1.0E-7) and *ibm1-6* (7.4 ± 1.4; n = 30; p-value = 1.5E-8) had significantly fewer foci (Fig 5D and 5E), indicating that IBM1 is required to produce WT level of type I COs.

To further examine which region of chromosome reduces COs, we used the previously described Fluorescent Tagged Lines (FTL) system [46] to measure CO frequency in 5 genetic intervals on 3 different chromosomes (S5A Fig). The genetic distances in I2a/b interval significantly decreases from 2.95 ± 0.559 cM and 8.03 ± 0.401 cM in WT to 2.15 ± 0.186 cM (p-value = 0.034) in *ibm1-4* and 4.10 ± 0.485 cM (p-value = 0.00013) in *ibm1-6* (S5B Fig), respectively. In contrast, the genetic distances in the intervals of I3 and I5 significantly increase from 7.19 ± 0.795 cM in WT to 12.6 ± 1.20 cM (p-value = 0.0011) across I3c in *ibm1*, and from 2.76 ± 0.573 cM and 4.61 ± 0.421 cM (WT) to 5.52 ± 1.43 cM (p-value = 0.027) and 8.81 ± 0.91 cM (p-value = 0.0013) across I5c and I5d in *ibm1-6* (S5B Fig). We then calculated the interference ratio (RA) of I2ab and I5cd, and found that RA of I2ab showed no significant difference (p-value = 0.079, one-tailed student t-test) with 0.15±0.13 (n = 2081) in WT compared with 0.67±0.64 (n = 1924) in *ibm1-6*. In contrast, RA of I5cd significantly decreases (p-value = 0.044, one-tailed student t-test) with 0.60±0.14 (n = 2310) in WT in comparison to 0.72±0.16 (n = 2300) in *ibm1-6*. These results suggest that IBM1 promotes CO formation likely via affecting CO interference or distribution. The discrepancy between the HEI10 and FTL assays may reflect the fact that HEI10 foci are measured in diakinesis meiocytes while the FTL system assays mature pollen grains and meiosis with too few COs may fail to mature. Alternatively, it may be because the HEI10 assay measures only Type I COs while the FTL assay measures both Type I and Type II COs. Finally, the FTL assay may be detecting interval-specific phenotypes while the HEI10 assay is measuring a global phenotype.

### IBM1 localizes on early meiotic prophase I chromosomes

**Fig 6:**
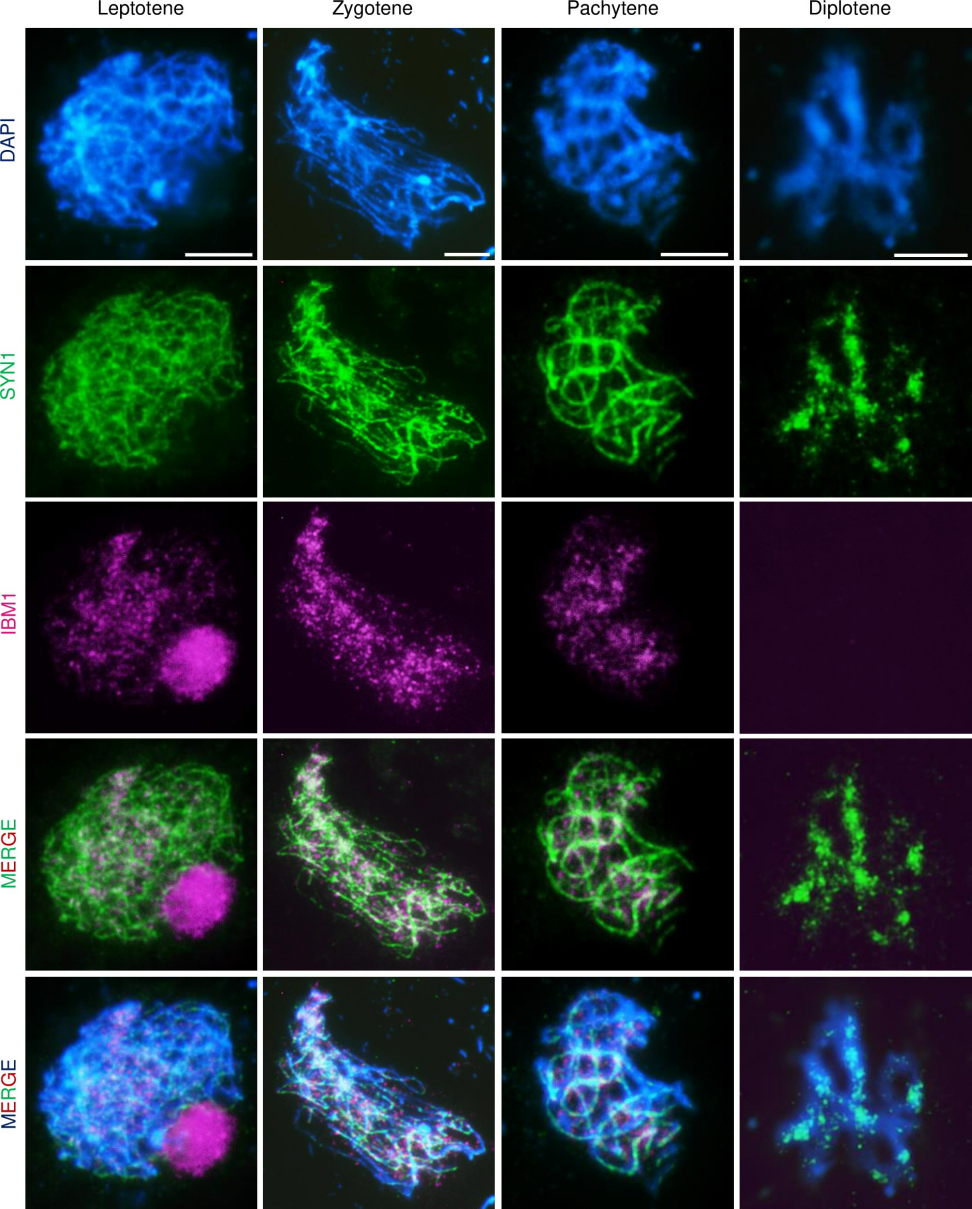
Immunofluorescence of IBM1 in the *IBM1* trans-complemented plants at leptotene, zygotene, pachytene and diplotene. Dual-immunofluorescence images with anti-FLAG antibody (magenta) and an antibody against the meiosis-specific cohesin protein SYN1 (green). An IBM1-FLAG tag fusion was expressed in *ibm1-6* plants and IBM1 can be observed colocalizing with chromosomes (DAPI, blue) at leptotene, zygotene, pachytene but not diplotene. Scale bar: 5 μm.

**Fig 7:**
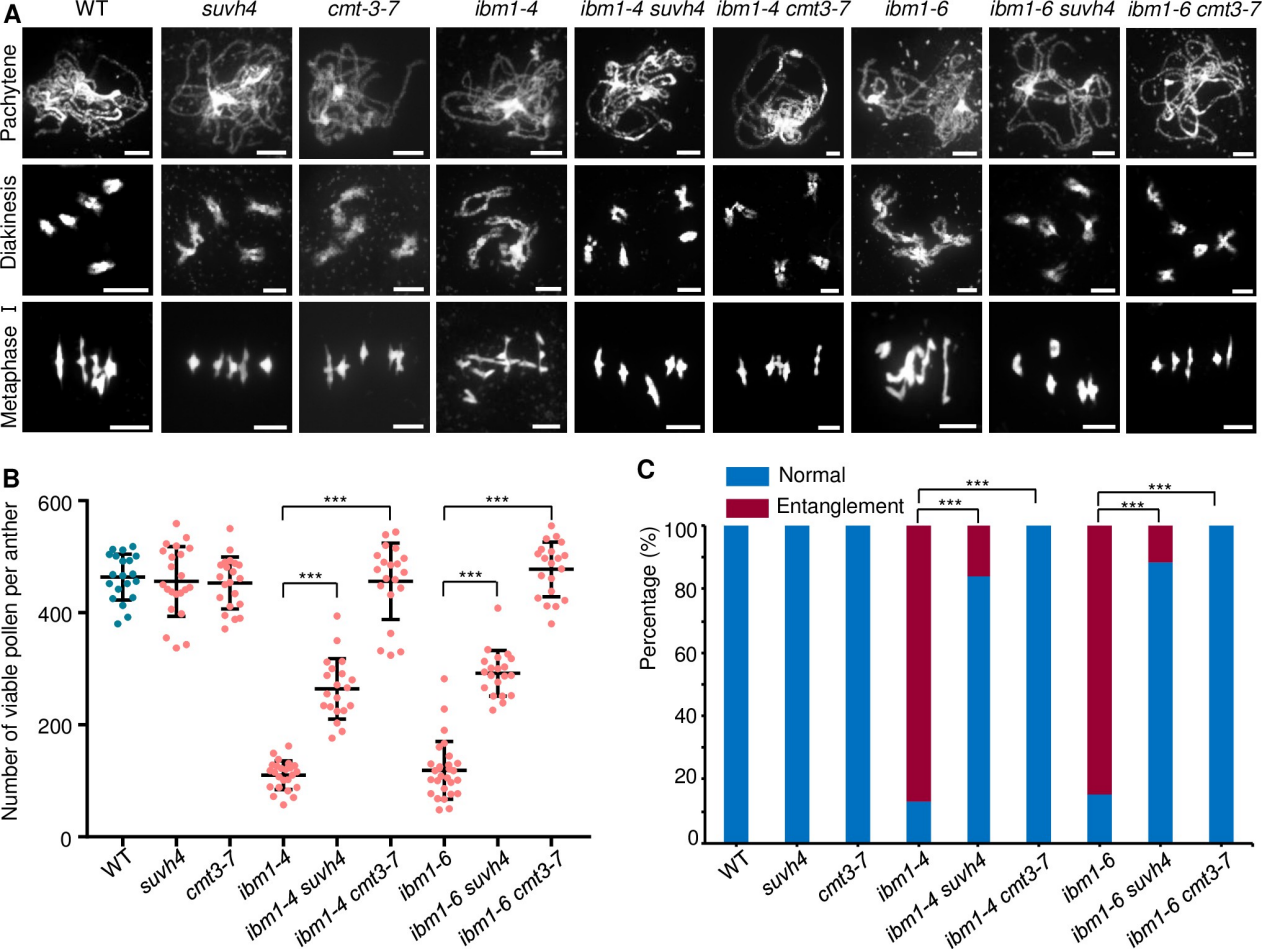
*suvh4*/*kyp* and *cmt3* suppress meiotic defects of in *ibm1*. (A) DAPI stained pachytene (top row), diakinesis (middle row) and metaphase I (bottom row) chromosomes from WT, *ibm1-4*, *ibm1-6*, *suvh4*, *cmt3*, *ibm1-4 cmt3*, *ibm1-4 suvh4*, *ibm1-6 cmt3* and *ibm1-6 suvh4* meiocytes. Scale bar: 5 μm. (B) Quantification of viable pollen grains per anther from WT, *ibm1-4*, *ibm1-6*, *suvh4*, *cmt3*, *ibm1-4 cmt3*, *ibm1-4 suvh4*, *ibm1-6 cmt3* and *ibm1-6 suvh4* plants. *** represents p-value< 0.001 in two-tailed student *t* test. The data are shown as mean ± SD. (C) Image showing percentage of chromosome with (entanglement) or without (normal) entanglement in Metaphase I. *** represents p-value< 0.001 in two-tailed student chi-square test.

*Arabidopsis* IBM1 was previously reported to demethylate H3K9me2 in somatic cells [27]. We used an *in vivo* transient expression assay with *IBM1* with a C-terminal *GFP* fusion expressed by the 35S promoter (*p35S*::*IBM1*-*GFP*) in *Nicotiana benthamiana* leaves coupled with immunofluorescence staining of methylated histone H3K4me1/2, H3K9me2 and K27me1. By comparing the relative intensity of methylated histone H3 in the nuclei with or without *p35S*::*IBM*-*GFP*, H3K9me2 demethylation can be measured [47]. Our results show that IBM1 does not demethylate H3K27me1, H3K4me1 or H3K4me3, but is able to demethylate H3K9me2 (S6 Fig), consistent with pervious findings [27]. To test whether IBM1 also has a demethylase activity in meiocytes, we examined IBM1 localization using an anti-FLAG antibody in meiocytes from *ibm1-6* plants that had been transformed with a *pIBM1*::*IBM1-FLAG* rescue construct. Using dual-immunofluorescence with an antibody against the meiosis-specific cohesin protein SYN1, we detected FLAG signal that primarily overlaps chromosomes in leptotene, zygotene and pachytene meiocytes. Intriguingly, the FLAG signal is no longer associated with chromosomes after pachytene (Fig 6). The localization of IBM1 on early meiotic chromosomes suggests activity during meiosis. We used immunofluorescence to detect H3K9me2 (using H3K4me3 as a control) in WT and *ibm1* meiocytes. H3K9me2 is mainly localized at heterochromatic regions in WT zygotene meiocytes (S7A Fig) and has equivalent fluorescence intensities in WT, *ibm1-4* and *ibm1-6* (S7C Fig). At pachytene, the fluorescence intensity of WT H3K9me2 is significantly reduced (p-value = 0.043) (S7C Fig), whereas the *ibm1* mutant continues to maintain similar levels of H3K9me2 signal compared to zygotene (Figs 7C and S7B). By comparison, the fluorescence intensity of the H3K4me3 control does not differ between zygotene and pachytene or between WT and *ibm1* (S7D–S1F Fig). These results suggest that IBM1 demethylates H3K9me2 during meiotic prophase I.

### Meiotic defects in *ibm1* are rescued by loss of either *SUVH4/KYP* or *CMT3*

In *Arabidopsis* somatic cells, H3K9 dimethylation by SUVH4/KYP and DNA CHG methylation by CMT3 form a regulatory loop [23,48], and the *ibm1* fertility defects can be restored by either *cmt3* or *suvh4/kyp* [26]. If this also takes place in meiocytes, we hypothesized that simultaneous mutation of *IBM1* and either *SUVH4/KYP* or *CMT3* could rescue the meiotic defects in *ibm1*. To test this idea, we crossed *ibm1-4* and *ibm1-6* with *cmt3* and *suvh4*/*kyp* mutants. Single *suvh4*/*kyp* or *cmt3* mutants have pollen viability (Figs S8A and 7B) and chromosome morphology phenotypes (Fig 7A) that are indistinguishable compared with WT. The *ibm1 cmt3* and *ibm1 suvh4* double mutants have phenotypes that resemble the single *suvh4*/*kyp* or *cmt3* mutants including restored male fertility and meiotic chromosome morphologies (Figs 7 and S8), but the degree of restoration in the *cmt3* background is better than *suvh4* (*ibm1-4 cmt3* vs. *ibm1-4 suvh4*: p-value = 0.045; *ibm1-6 cmt3* vs. *ibm1-6 suvh4*: p-value = 0.022; n>14) (Figs 7C and S8C). These results support the idea that the H3K9me2-CHG methylation regulatory loop also functions in meiocytes. To further validate this model we examined *in vivo* DNA methylation in *ibm1* using an immunofluorescence assay with an antibody against 5mC combined with FISH using a centromeric DNA probe. In WT meiocytes 5mC methylation is strongest in the pericentromeric regions (S9A and S9B Fig), and shows a gradual reduction from zygotene to pachytene. By contrast, the 5mC signals persist in *ibm1* during the transition from zygotene to pachytene (S9C Fig). These results suggest that IBM1 influences DNA methylation during meiosis.

### Mutation of *IBM1* causes genome-wide elevation of genic CHG methylation

**Fig 8:**
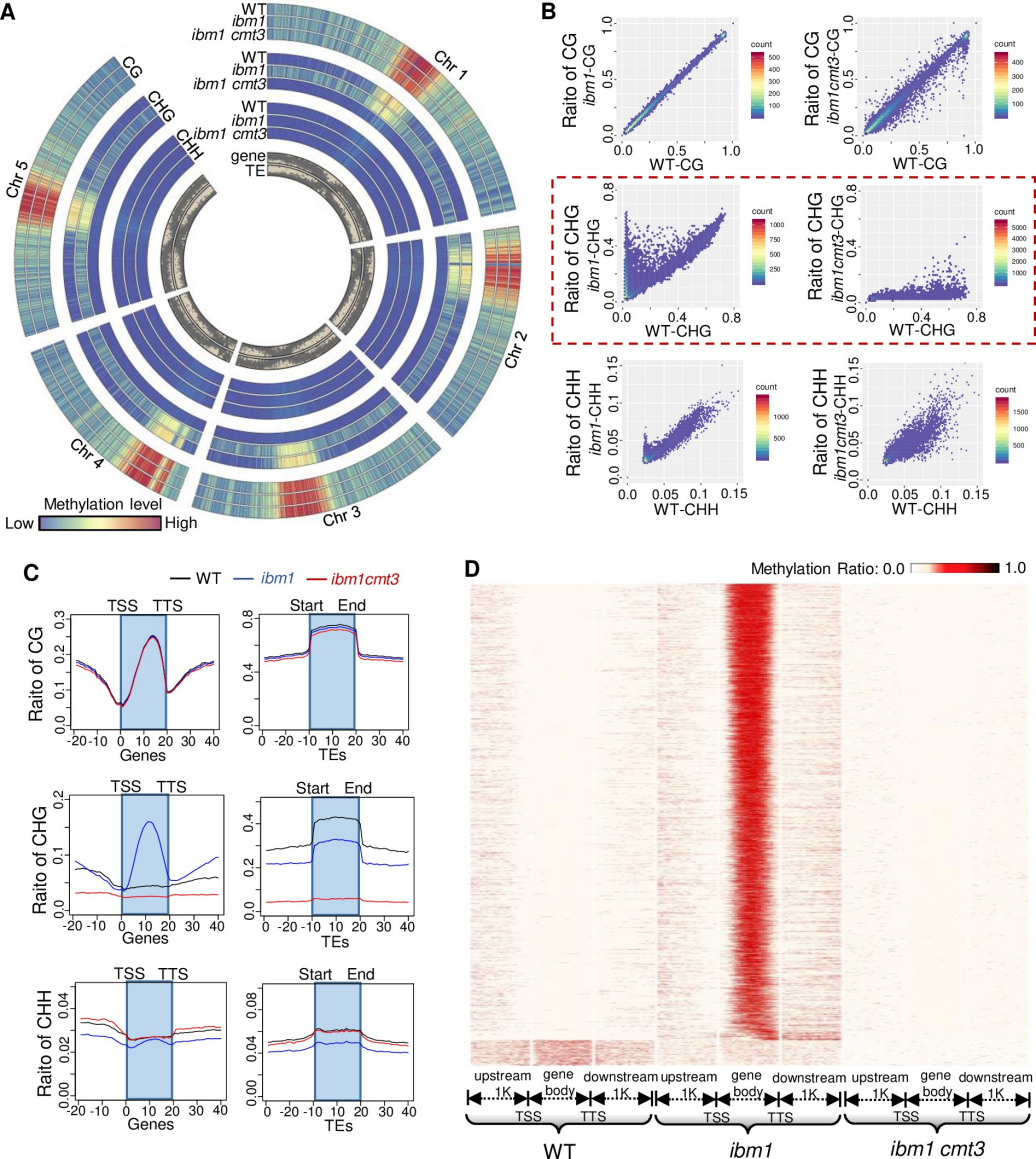
IBM1 is required for gene body CHG demethylation. (A) The relative CG, CHG and CHH methylation levels along the 5 Arabidopsis chromosomes is shown as a heat map. Each type of DNA methylation was measured in WT (the outside lane), *ibm1-6* (the middle lane) and *ibm1-6 cmt3-7* (the inside lane). CHG methylation is elevated (redder colors) in *ibm1*, and reduced in *ibm1 cmt3* (bluer colors). The distribution of genes and transposon elements (TEs) is shown in grey (inner rings). (B) Scatter diagram showing CG, CHG and CHH methylation ratios in WT, *ibm1-6* and *ibm1 cmt3*. The red dotted line highlights CHG methylation. (C) The distribution of CG, CHG and CHH methylation level along genes, with gene bodies flanked by transcriptional start sites (TSS) and transcriptional termination sites (TTS), and TEs. (D) Heat map showing distribution of CHG methylation in genes with elevated CHG methylation within gene body in WT, *ibm1-6* and *ibm1 cmt3*.

To more precisely measure genome-wide DNA methylation changes caused by mutation of *IBM1*, we performed whole genome bisulfate sequencing (WGBS) of *ibm1-6* (segregated from *ibm1*^*-/+*^ heterozygotes) stage 4–7 anthers that contain meiocytes spanning all stages of meiosis. Our results show that euchromatic instead of heterochromatic regions in *ibm1* have higher CHG methylation compared to WT, while CG and CHH methylation are not significantly different (Fig 8A and 8B). Examination of CG/CHG/CHH methylation in the region in and around genes shows that CHG methylation is elevated from transcription start sites (TSS) to transcription termination sites (TTS) in *ibm1* compared to WT (Fig 8C and 8D). In contrast, CHG methylation in transposons decreased in *ibm1* compared with WT, consistent with prior analysis in *ibm1* somatic tissues [49] (Figs 8C and S10). We classified TEs into three categories based on their distance from genes, TE in intragenic regions, those in 2kb flanking regions of genes and far from genes (>2kb). TEs of the “>2kb” category exhibit significant decrease in *ibm1* for most of retrotransposons and DNA transposons, followed by TEs of the “<2kb” category and by “intragenic” TEs. These results suggest that IBM1 is influencing DNA methylation patterns in meiocytes similar to its action in mitotic tissues [29,49]. To further characterize the rescue of meiotic defects in *ibm1-6* by loss of CHG methyltransferase *CMT3* we described earlier (Fig 7), we compared the DNA methylation patterns of the *ibm1-6* single mutant and *ibm1-6 cmt3-7* double mutant at a whole genome scale. In the *ibm1-6 cmt3-7* double mutant, the elevation of euchromatic CHG methylation observed in the *ibm1-6* single mutant was eliminated (Fig 8A and 8B). Consistent with our prior observations, the restoration of WT-like CHG methylation patterns is mainly attributable to changes within gene bodies (Fig 8C and 8D). It is noteworthy that stage 4–7 anthers contain both somatic and meiotic cells, thus limiting the resolution with which CHG methylation can be detected. Future studies may benefit from the use of single-cell sequencing approaches.

### IBM1 regulates gene expression in meiocytes

**Fig 9:**
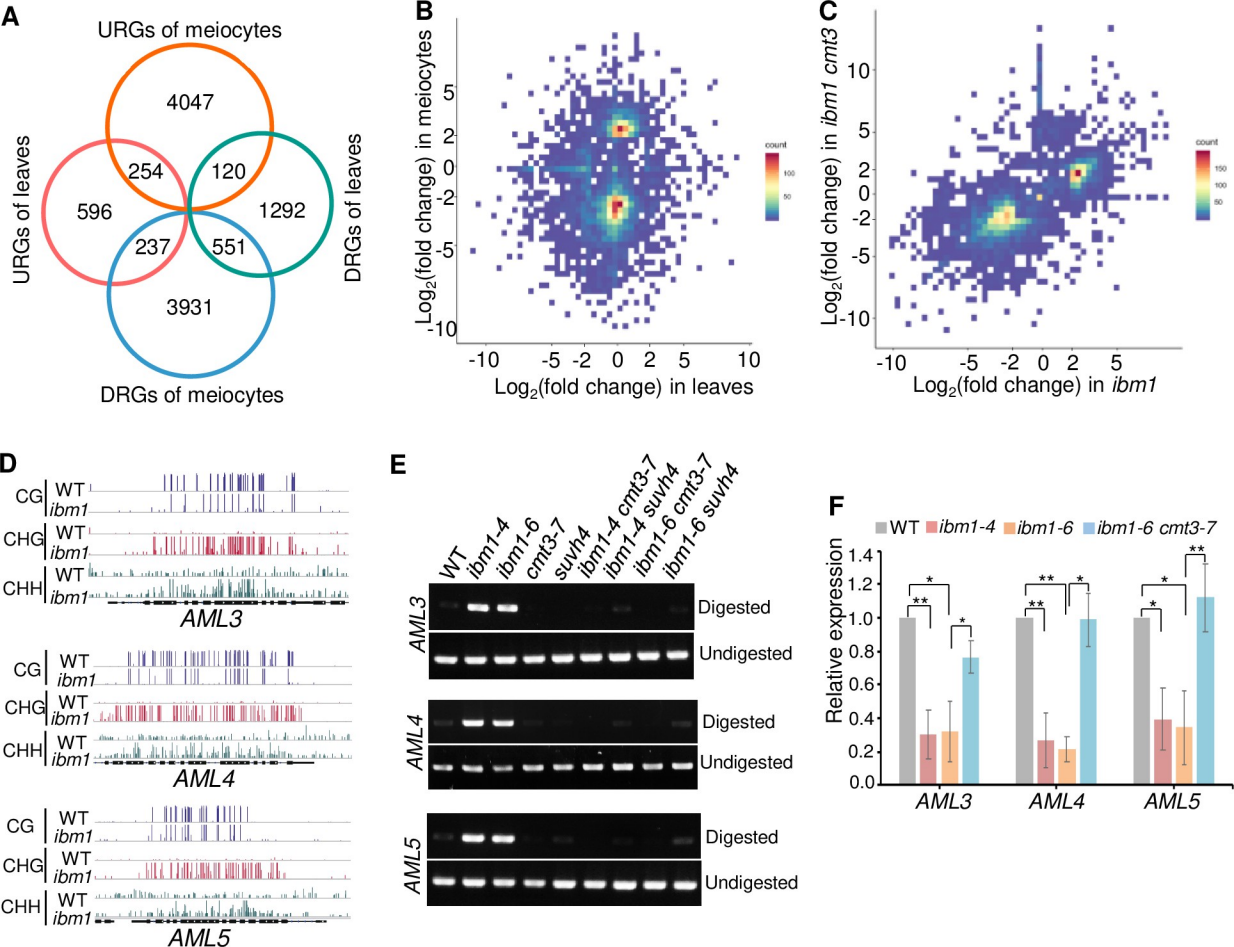
IBM1 specifically regulates many genes expressed in meiocytes. (A) Venn diagram showing genes with significantly reduced or increased expression in *ibm1-4* meiocytes and leaves. (B) Diagram showing Log_2_(fold change) in *ibm1-4* meiocytes and leaves. Red rectangles indicate genes specifically downregulated in meiocytes. (C) Diagram showing Log_2_(fold change) in *ibm1* meiocytes and *ibm1 cmt3* meiocytes. Red rectangles indicate genes with restored expression in *ibm1-6 cmt3-7* meiocytes. (D) Distribution of CG (top rows), CHG (middle rows) and CHH (bottom rows) methylation within gene body of *AML3* (top row), *AML4* (middle row) and *AML5* (bottom row) in 4–7 stage anthers of WT and *ibm1*. (E) CHOP-PCR analysis of *AML3-5* in 4–7 stage anthers of WT, *ibm1-4*, *ibm1-6*, *cmt3*, *suvh4*, *ibm1-4 cmt3*, *ibm1-4 suvh4*, *ibm1-6 cmt3* and *ibm1-6 suvh4*. Genomic DNA was digested by MSP1, which recognizes the CCGG site. Methylation of the first C prevents cutting by MSP1. (F) RT-qPCR analysis of *AML3-5* expression in meiocytes of WT, *ibm1-4*, *ibm1-6* and *ibm1-6 cmt3-7*. * p-value<0.05, ** P-value<0.01 with two-tailed student *t* test. The error bars represent the SD of each group.

IBM1-dependent gene-body demethylation regulates the expression of specific genes such as *ERECTA* family genes in somatic cells [30]. To differentiate loci that are regulated by IBM1 in somatic cells and meiocytes, we performed RNA-seq using 3-week-old leaves (as control) and isolated meiocytes from WT and homozygous *ibm1* segregated from *ibm1*^*-/+*^ heterozygotes. Compared with WT, we found significantly more differentially expressed genes (DEGs) in meiocytes (9,140; 2 folds change and p-value< 0.01) than did in leaves (3,050) (Figs 9A and S12). 4,421 DEGs were up-regulated and 4,719 down-regulated in meiocytes compared to 1,087 and 1,963 up-/down-regulated DEGs in leaves. Moreover, 4,168 (94.3%) and 4,167 (88.3%) genes were specifically up- and down-regulated in *ibm1* meiocytes, suggesting that IBM1 has distinct targets in meiocytes as compared to leaves (Fig 9A and 9B). We performed gene ontology analysis on the genes that are specifically up-/down-regulated in leaves (833 up and 1,412 down) or meiocytes (4,167 up and 4,168 down), and found that meiocyte-specific down-regulated genes (DRGs) were enriched in different biological processes related to reproductive development (GO:0061458) (S13A Fig), including known meiotic genes such as cohesion associated proteins SYN4 and WAPL2 [50]. In particular, among five members of the *AML* family (*AML1*-*5*) [51], three (*AML3*-*5*) have 3–5 fold lower expression in *ibm1* meiocytes compared to in WT (S3 Table). Intriguingly, the 4,167 meiocyte-specific up-regulated genes (URGs) are enriched in multiple meiotic processes, such as homologous recombination (GO:0035825) (S13A Fig), including ZIP4 and DMC1 which function during synapsis and strand-invasion respectively [5]. These results show that IBM1 affects the expression of different genes in leaves and meiocytes, but has a more prominent role in the latter.

Given that mutation of *CMT3* suppresses the meiotic defects in *ibm1*, we also sequenced the transcriptome of *ibm1-6 cmt3-7* double mutant meiocytes. The total number of DEGs in *ibm1 cmt3* was dramatically lower than that in *ibm1* with 804 up- and 1,879 down-regulated DEGs in *ibm1 cmt3*. Interestingly, the expression of 2,269 up-regulated genes in *ibm1* was restored to close to normal levels in *ibm1 cmt3* (Fig 9C). Surprisingly, genes involved in multiple meiotic processes were not restored, suggesting that the expression upregulation of those genes in *ibm1* is independent on CMT3, similarly, 1,834 down-regulated DEGs in *ibm1* were also restored in *ibm1 cmt3* (Fig 9C) and were mainly enriched in reproductive development (GO: 0061458) and regulation of transcription (GO: 0045893) GO terms (S13B Fig). We further focused on the expression of 128 meiotic known genes in *ibm1* and *ibm1 cmt3*. Consistent with GO analyses (S13A and S13B Fig), 84 genes were significantly up-regulated in *ibm1* mutant and 74 out of 84 genes did not have restored expression in *ibm1 cmt3*, including genes involving DSB repair and synapsis, indicating that the meiotic abnormalities in *ibm1* are not caused by upregulation of these genes (S14 Fig and S3 Table). Then we asked whether the meiotic defects in *ibm1* are resulted by genes with downregulation. We compared these 1,834 down-regulated DEGs with our gene body CHG methylation data, and identified 437 genes that also have significantly elevated CHG methylation (S14D–S14F Fig). GO enrichment analysis of these 437 genes found that they are enriched in reproductive development and regulation of transcription (S13C Fig). We speculate that those 437 genes may contribute to the meiotic function of IBM1.

Interestingly, the expression of *AML3*, *AML4* and *AML5* are significantly rescued in *ibm1 cmt3*, especially *AML5* (S3 Table). The gene body DNA methylation level of anthers and expression level of *AML3-5* in meiocytes were further validated by CHOP-PCR and RT-qPCR respectively (Fig 9D–9F). In contrast, although gene body DNA methylation patterns in 3-week-old leaves of *AML3*, *AML4* and *AML5* in *ibm1*, *ibm1 suvh4* and *ibm1 cmt3* mutants are similar to that in anthers or meiocytes (Figs 9D, 9E, S15A and S15B), the expression level of *AML3*-*5* show no significant change in *ibm1* leaves (S15C Fig and S3 Table). These results suggest that IBM1 specifically affects the expression of *AMLs* in meiocytes instead of leaves.

**Fig 10:**
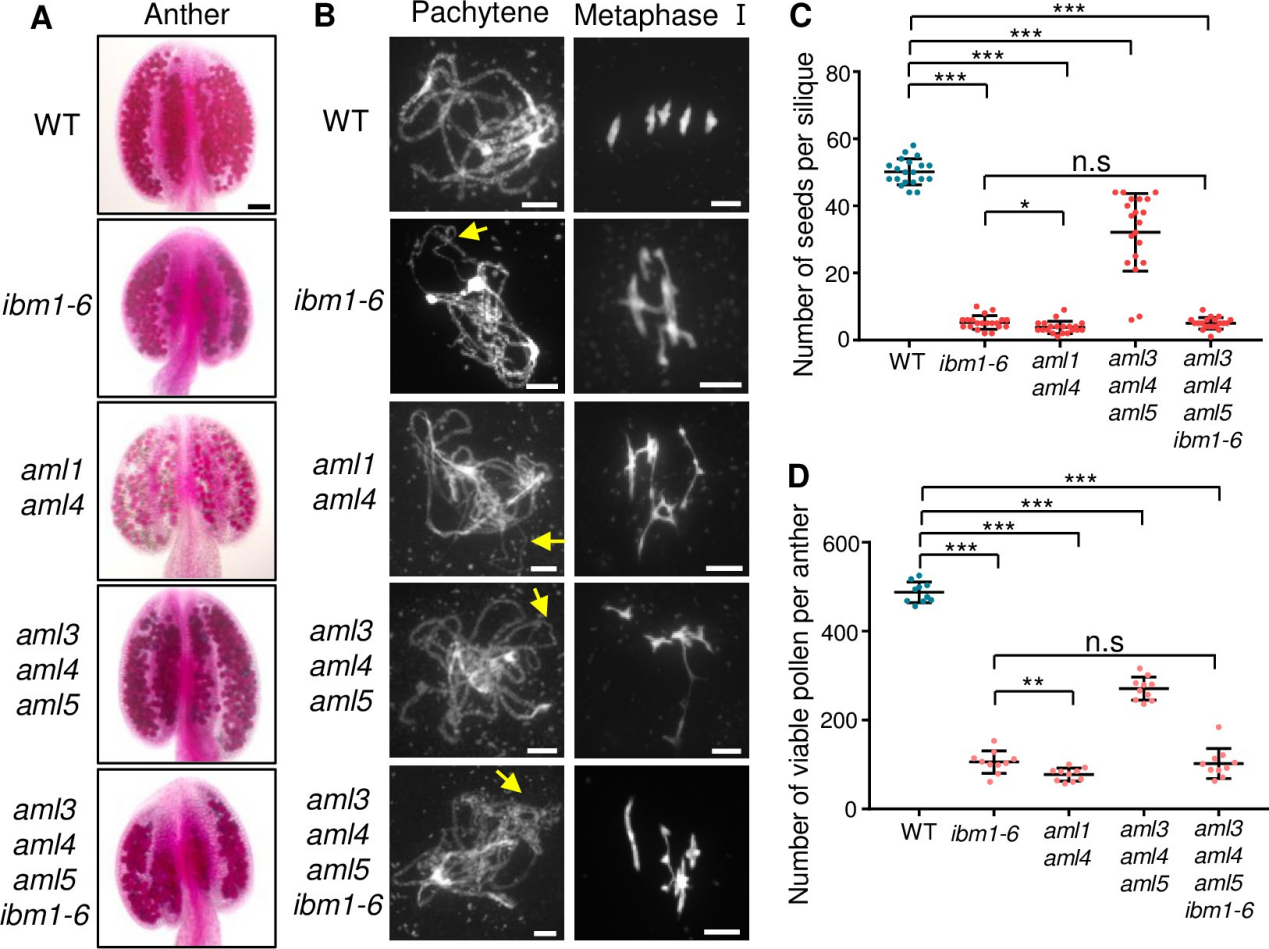
Mutation of *AML* family reduces fertility and causes similar meiotic defects with *ibm1*. (A) Alexander staining of anthers in WT, *ibm1-6*, *aml1 aml4*, *aml3 aml4 aml5* and *aml3 aml4 aml5 ibm1-6*. (B) Chromosome spreads showing meiotic chromosome morphologies of WT, *ibm1-6*, *aml1 aml4*, *aml3 aml4 aml5* and *aml3 aml4 aml5 ibm1-6* meiocytes. Yellow arrows on mutant pachytene chromosomes indicate the unsynapsed regions. (C) Quantification of viable pollen grains per anther of WT, *ibm1-6*, *aml1 aml4*, *aml3 aml4 aml5* and *aml3 aml4 aml5 ibm1-6*. *** represents p-value< 0.001 in two-tailed student *t* test. The data are shown as mean ± SD. Scale bars: A: 50 μm, B: 5 μm.

*Arabidopsis AML* genes were previously shown to be functionally redundant in meiosis and the *aml1 aml2 aml4* triple mutant has multiple meiotic defects in synapsis and recombination [51], similar to those observed in *ibm1* (Fig 2). Therefore, we hypothesize that the *ibm1* meiotic defects may be caused, at least in part, via the reduced expression of one or more *AML* genes. To test this hypothesis, we analyzed *aml3 aml4 aml5* triple mutant, which show significant reduction of pollen viability compared with WT (Fig 10A, 10C and 10D). Furthermore, *aml3 aml4 aml5* meiocytes exhibit uncomplete synapsis and chromosome entanglement similar to *ibm1* (Fig 10B). We also observed that *aml1 aml4* double mutant has severe defects in male fertility and meiosis than that of *aml3 aml4 aml5* (Fig 10), similar to previous report of *aml1 aml2 aml4* triple mutant [51]. Genetic analysis of *aml3 aml4 aml5* with *ibm1-6* found that the fertility and the meiotic defects of *aml3 aml4 aml5 ibm1-6* resembles *ibm1-6* (Fig 10), suggesting that AMLs may function on the same pathway as IBM1 during meiosis.

Because IBM1 affects majority of genes including *AML* family, we attempted to introduce *AML5* expressed by the *Actin 7* promoter (*pAct7*) [52] into *ibm1-6* plants. We obtained totally 39 transformed positive T1 plants and further selected 20 independent lines for the subsequent study. According to silique length and seed number, 12 plants have significantly elevated fertility (S4 Table and S16A Fig). Alexander staining shows that 9 plants have significantly elevated number of viable pollen grains (S16B and S16C Fig). We further chose two lines with well restored (#19 and #20) fertility and level of AML5 protein (S16E Fig) for chromosome spread analysis. The proportion of incomplete synapsis in #19 (9/26; p-value = 0.048) and #20 (13/36; p-value = 0.012) (S16D Fig) and chromosome entanglement in #19 (4/9; p-value = 0.016) and #20 (7/16; p-value = 0.018) (S16D Fig) are significantly reduced compared with *ibm1-6*. Together, these results suggest that the meiotic defects observed in *ibm1* mutants are at least partially AMLs-dependent.

## Discussion

### Normal meiotic progression requires the H3K9me2 demethylation but not methylation

Chromatin contains euchromatic and heterochromatic regions that have distinct epigenetic marks such as enrichment of H3K4me3 in euchromatin and H3K9me2 in heterochromatin [53]. H3K4me3 is correlated with meiotic CO hotspots in animals and plants [12,13,15,18], while H3K9me2 is negatively correlated with meiotic COs [11]. In *Arabidopsis*, H3K9me2 is mainly generated by SUVH4/KYP, SUVH5 and SUVH6 [7], but *suvh4/5/6* triple mutant does not have obvious meiotic defects [54]. Nonetheless, the number of DSBs and COs is significantly elevated at pericentromere regions in *suvh4/5/6* [11], suggesting that establishment of H3K9me2 is dispensable for meiosis progression, but has a role in shaping CO distribution. Here we provided strong evidence to support the idea that normal meiosis progression requires removal of H3K9me2. We show that IBM1 which is known to demethylate H3K9me2 in somatic cells [26,27,29], also localizes on chromosomes at early meiotic prophase I (Fig 6). Immunostaining data shows a significant increase of H3K9me2 on pachytene chromosome in *ibm1* compared with WT. These results suggest that IBM1 is functioning as an H3K9me2 demethylase during meiosis. Consistent with this, we show that *ibm1* mutants have meiotic defects in synapsis and recombination that result in reduced fertility. Our transcriptomic analysis has also shown that IBM1 regulates significantly more genes in meiocytes than leaves including those that mediate critical meiotic processes. Based on these observations, we hypothesize that successful pairing, synapsis and recombination of homologous chromosomes during meiotic prophase I requires regional modification of chromatin including removal of histone marks to ensure appropriate gene expression. This is consistent with prior findings that the H3K4me3 demethylase JMJ16 is required for meiotic chromosome condensation by mediating specific gene expression [24]. Our data extends this model to include the H3K9me2 demethylase IBM1 as a critical regulator of meiotic gene expression.

### Conservation and divergence of H3K9 demethylation in meiosis between plants and mammals

In mammals, H3K9me3 is a heterochromatin enriched marker. Heterochromatin in plants, by comparison, is less enriched in H3K9me3 and is instead characterized by high levels of H3K9me2 [53]. Mutation of the mouse H3K9me3 methyltransferases, *Suv39h1* and *Suv39h2*, cause severe meiotic defects, including delayed synapsis and nonhomologous chromosome interaction [55]. However, disruption of the testis-specific H3K9me3 demethylase *KDM4D* leads to a dramatic increase of H3K9me3 signals, but does not affect meiosis and fertility in mice [56], suggesting that H3K9me3 methylation rather than demethylation is important for meiosis in mice. We have found that *Arabidopsis* IBM1 is critical for meiosis and regulates the expression of a large set of genes specifically in meiocytes. Interestingly, these changes in gene expression are also correlated with changes in gene body CHG DNA methylation, which is consistent with previous studies of IBM1 activity in somatic cells [30,57]. Transposons did not appear to be similarly affected by IBM1, which is also consistent with previous findings in somatic cells [49]. In contrast, mouse JHDM2A (JMJC DOMAIN CONTAINING HISTONE DEMETHYLASE 2A) is the ortholog of *Arabidopsis* IBM1 and demethylates H3K9me2 enriched in mammalian euchromatin [58,59]. During meiosis, JHDM2A localizes to chromosomes at pachytene and directly regulates the expression of both *Tnp1* (Transition nuclear protein 1) and *Prm1* (Protamine 1), and is important for controlling spermatogenesis post meiotically [60]. These results suggest that IBM1 has similar biochemical activities in plants and animals, and regulates genes expression in both lineages by modulating the epigenetic landscape, but has diverges in the specific meiotic function in controls in animals and plants.

### IBM1 mediates distinct gene expression between leaves and meiocytes

Our results demonstrated that regulation of gene expression by the IBM1-mediated removal of H3K9me2 and its secondary influence on gene body CHG methylation is conserved between somatic cells and meiocytes in plants. However, IBM1 appears to affect many more genes in meiocytes as compared to leaves. The expression of over 88% of *Arabidopsis* genes are altered in meiotic cells compared with only 71% leaves demonstrating that IBM1 has specific meiotic targets that are distinct from those in somatic tissues. This raises the question of how IBM targets specific loci. IBM1 has two putative RING domains and a JmJC domain. RING domains appears to lack E3 ligase activity [34], and the RING domain’s function is unknown. It is possible that the IBM1’s RING domains may help mediate its association with chromatin, thus determining its target specificity. Alternatively, IBM1 may be recruited by an interacting partner such as the JMJ16-MMD1 module [24]. Another question raised by our observations is why IBM1 appears to regulate many more genes in meiocytes as compared to somatic tissues? It is plausible that during early meiotic prophase I, prior to formation of double strand breaks (DSBs), on the accessibility of chromatin needs to be altered to a more open state which is facilitated by IBM1’s removal of H3K9me2 which in turn influences CHG methylation. As reported previously, sites associated with crossovers are often in transcriptional active regions which suggest a correlation with open chromatin. However, with the present data, we cannot preclude that IBM1 is exerting its influence in meiocytes through the secondary effects of the genes it transcriptionally regulates.

### The role of IBM1 in meiosis

Previous findings and our data support the idea that the major role of IBM1 is to regulate gene expression via removal of H3K9me2 which in turn promotes CHG demethylation within gene body. In cells from somatic tissues such as leaves, IBM1 is known to regulate genes such as *ERECTA* during stomatal development [30] and *PR1* during plant-pathogen interactions [57]. In meiocytes, IBM1 targets many genes including *AML3-5*. *Arabidopsis aml1 aml2 aml4* triple mutants have multiple meiotic defects including incomplete synapsis and defects in DSB repair [51]. Here we found that meiotic phenotypes of *aml3 aml4 aml5* are tantalizingly similar to the phenotypes we see in *ibm1*. However, the function of the AML family of proteins in plant meiosis is unclear. In *Schizosaccharomyces pombe*, *Mei2*, the *AML* homologous, is a master regulator of meiosis. Mei2p promotes premeiotic DNA synthesis and interacts with another RNA binding protein Mmi to prevent meiotic transcript degradation [61]. Mei2p has three RNA recognition motifs (RRMs), which are conserved in AMLs. Thus, it is possible that *Arabidopsis* IBM1’s regulation of AML transcription in meiocytes may also influence the RNA degradation machinery.

In summary, IBM1 was first identified as a H3K9me2 demethylase which mainly regulated the epigenetic architecture of gene body regions including euchromatic gene-like TEs but not transposons [26–29,31], and its regulation of gene expression influences multiple biological processes [30,57]. Here we demonstrate that IBM1 regulates a broad array of target genes during meiosis, including *AMLs* and is required for normal meiotic progression. These results significantly advance our understanding of IBM1 role in reproductive development.

## Materials and methods

### Plant materials and growth condition

Dr. Xiaofeng Cao (Institute of Genetics and Developmental Biology, Chinese Academy of Sciences) provided *ibm1-6* (SALK_006042). The other mutants of *ibm1*-*4* (SALK_035608), *suvh4*/*kyp* (SALK_069326), *cmt3-7* (point mutation), *aml1* (SALK_015088), *aml3* (SALK_006041), *aml4* (SALK_019467), *aml5* (SALK_061664), *msh4* (SALK_136296), *mus81* (SALK_107515) and *spo11-1* [40] were obtained from the Arabidopsis Biological Resource Center (http://www.arabidopsis.org). The *ibm1* and its double mutants used for analysis of pollen viability, tetrad, chromosome spread, FISH, immunostaining, CHOP-PCR, RNA-seq and BS-seq, were segregated from the first generation of heterozygous *ibm1*^*+/-*^plants. Double mutants were generated by crossing heterozygous single mutants and selecting appropriate genotypes from the resulting F2 populations. Mutant genotypes were confirmed by sequencing using specific primers listed in S4 Table. All genotypes except for *cmt3-7* (Landsberg, Ler background) and *spo11-1* (Wassilewskija, WS background) are Col-0 background. All WT, mutants and transgenic plants were grown at 21°C in a greenhouse under long-day conditions (16h light/8h dark).

### Alexander and toluidine blue staining

Alexander staining was performed as described previously with minor adjustments [62]. Briefly, anthers were incubated with Alexander staining solution for 30 min (65°C). Tetrad-stage microspores were stained with toluidine blue dye as previously described [63]. Images of tetrad and mature pollen were collected using a Zeiss Axio Scope A1 microscope (Zeiss, Germany).

### Plasmid construction and plant transformation

For trans-complementation testing of *ibm1-6*, the full-length coding sequence of IBM1 was amplified using primers with BamH I and Sal I restriction enzyme sites added at each end. 1166 bp of DNA upstream of the IBM locus ATG start codon was amplified using primers with EcoR I and Sac I sites at each end, and used as the native IBM1 promoter. The IBM1 coding region and its promoter were cloned into the pCAMBIA 1306 plasmid upstream of *3×FLAG* [64], and transformed into *Agrobacterium tumefacien* GV3101 and then transformed into *ibm1-6*^*+/-*^ plants. T1 seeds were screened on 1/2 MS (Murashige-Skoog) agar plates with 25 mg/L hygromycin for 12 days. The primers are listed in S4 Table.

### Chromosome spread, centromere FISH, immunostaining and chromosome painting

Chromosome spreading, centromere FISH and immunofluorescence assays were conducted following procedures described previously with some adjustments [63]. The primary antibodies, γ-H2AX (1:200), DMC1 (1:400), ASY1 (1:200), SYN1 (1:200) and HEI10 (1:400), were raised in rabbits and ZYP1 (1:100) was raised in rats [64]. The Alexa Fluor 488 Goat Anti-Rat IgG (A-21208; Invitrogen) and Alexa Fluor 555 Goat Anti-Rabbit IgG (A-21428; Invitrogen) secondary antibodies were diluted by 1:500 and 1:750, respectively. The colocalization images of ZYP1 and SYN1 and localization of ASY1 were captured using a Zeiss-LSM880 microscope. Other immunofluorescence images were obtained using a Zeiss Axio Scope A1 microscope. Chromosome spread and FISH images were captured using Zeiss Axio Scope A2 microscope.

The 5mC immunofluorescence assay combined with FISH using the 180bp centromeric repeat as a probe was done as previously reported with some adjustments [65]. Briefly, before adding primary and secondary antibodies, the slides were blocked by goat serum (BOETER, AR0009) for 20 min at room temperature. Primary antibodies were incubated for 30-40h at 4°C. Diluted FISH probes were added simultaneously with secondary antibodies and incubated for 2h at 37°C. After incubation, slides were washed using PBS with 0.1% Tween 20. Images were captured using a Zeiss Axio Scope A1 microscope.

The primary antibodies used were: Anti-Flag (Shanghai Genomics Technology, GNI4110-FG), anti-5mC (Abcam, ab10805) [66], anti-H3K9me2 (Abclonal, A2359) [67], and anti-H3K4me3 (Millipore, 07–473) [24]. Quantification was conducted by using Image J software and the integrated density subtracting background of each cell was utilized for measuring. The chromosome 1 painting, 5S and 45 S FISH are described previously [68].

### Measuring crossover frequency

Fluorescent Tagged Line (FTL) data collection, calculation of genetic distances and statistical analyses were performed according to Berchowitz & Copenhaver [46] and using the Stahl Lab Online tools (https://elizabethhousworth.com/StahlLabOnlineTools/).

### Quantitative RT-PCR and CHOP PCR

Male meiocytes were isolated using previously reported procedures and RNA was extracted using Trizol reagent (Invitrogen) [25]. Reverse transcription was conducted through using a PrimeScript RT with gDNA Eraser kit (Takara), and real-time PCR was performed using ChamQ Universal SYBR qPCR Master Mix (Vazyme, Q711-02) following the manufacturer’s instructions in a StepOnePlus real-time PCR system (Applied Biosystems).

For CHOP PCR, the genomic DNA of stage 4 to stage 7 anthers was extracted using cetyltrimethylammonium bromide (CTAB). [69]. 50 μL of anthers are collected in 1.5 mL tubes suspended in liquid nitrogen. 100ng of genomic DNA was digested with the methylation-sensitive restriction enzyme Msp I (Thermo, FD0544) in 20 μL reaction mix at 37°C for 75–90. Undigested genomic DNA was used as control. 0.5 μL of the digested DNA (or undigested control) was used as a template for PCR. Ethidium bromide (EB; v/v = 5%) was used to detect the PCR products after agarose gene electrophoresis (160V, 20 minutes). The primers are listed in S5 Table.

### *In vivo* histone demethylation assay

The full length coding sequence of IBM1 with an in-frame, C-terminal fusion of in- GFP expressed by the 35CaMV promoter was cloned into pCAMBIA 1306 plasmid. This construct was injected into half of a 4-week-old leaf of *Nicotiana benthamiana* [24], and the other half of the leaf was used as a control. Examination of the histone modification were performed as described previously [47]. To detect various epigenetic markers, the primary antibodies were used: H3K9me2 (ABclonal; A2359), H3K4me3 (Millipore; 07–473), H3K4me1 (Millipore; 07–436), H3K27me1 (Millipore; 07–448). Pictures were captured by using a Zeiss Axio Scope A1 microscope. The relative integrated intensity of 30 pairs of cells were quantified for each type of histone modification using Image J software. GFP-positive nuclei and nearby GFP-negative nuclei were selected in pairs for the analysis. The integrated intensity used for calculation excluded the background intensity of the same image.

### RNA-seq and analysis

For RNA-seq, we used two biological replicates for each sample. Meiocytes were collected following previously published procedures with minor adjustments [63]. At least 100 anthers were used for meiocytes collection in each biological replicate. Meiocytes most at zygotene and pachytene stage are isolated. Briefly, meiocytes were collected in RNase-free water mixed with Recombinant RNase inhibitor (2U/μL, Takara, 2313A) and 1/2 × DPBS (Thermo, 14190144). RNA was extracted as previous reported [25]. RNA libraries were constructed according to TruSeq RNA LT Sample Prep Kit v2 (Illumina). 2μg total RNA was used for subsequent procedures. Hiseq3000 with Hiseq3000SBS&Clusterkit were used for sequencing. The TAIR10 assembly of *Arabidopsis* Columbia genome and corresponding gene annotations were downloaded from the TAIR database [70]. Sequenced reads for wild type and mutant anthers were mapped against the reference genome using Bowtie2 v2.1.0 [71] and Tophat v2.0.9 [72], using annotated exons to perform transcript-guided mapping. DESeq2 [73] was used to quantify gene expression levels of reads within or spanning exons. We adopt the third-party software GFOLD to examine RPKM (Reads Per Kilobase per Million mapped reads) values of all genes and examined all DEGs under varying cutoffs of RPKM. The number of DEGs decreases slightly under low RPKMs, indicating that DEGs identified in *ibm1*are supported by sufficient sequencing depth. Genes with 2-fold or more difference in expression between mutants and wild type and with p-value lower than 0.01 were considered differentially expressed genes (DEGs). We applied a chi-square test with Bonferroni multi-test correction to control the false discovery rate (FDR) under 1%.

### Mapping of the Bisulfite-treated NGS reads and identification of methylation levels

For BS-seq, at least 200μL of stage 4–7 anthers were collected in 1.5mL tubes. DNA was extracted according to manufacturer’s instruction using the DNeasy Plant Mini kit (Qiagen, America). A total of 100ng genomic DNA was used to construct libraries with the EZ DNA Methylation Kit (Zymo Research). Novaseq6000 (Illumina) platform was used for sequencing. Image analysis and base calling were performed with Illumina CASAVA pipeline to produce 150bp pair-end reads. Trimmomatic v0.39 [74] software was used to trim and filter Bisulfite-treated short reads using the following parameters: “ILLUMINACLIP:TruSeq3-PE.fa:2:30:10 LEADING:3 TRAILING:3 SLIDINGWINDOW:4:15 MINLEN:100”. Clean reads were mapped onto the *Arabidopsis thaliana* reference genome sequences (TAIR10, http://www.arabidopsis.org) using BSMAP v2.90 [75]. Potential PCR duplicates were removed by using Sambamba v0.70 [76], reads with mapping qualities over 30 were kept for further analyses. Methylation ratios at each CG/CHG/CHH locus were measured using the Python script “methratio.py” in BSMAP [75].

Methylation levels were calculated for each gene/transposable element to facilitate comparison between WT and mutant plants under different sequence contexts. Fisher’s exact test was performed on the methylation ratios of a gene/transposable element to assess the statistical significance of the methylation level differences among samples. Specifically, a differentially methylated gene was defined when p-values of the Fisher’s exact test was smaller than 0.01 and the change of methylation level was over 20%.

## Supporting information

S1 Fig IBM1 is required for fertility. (A) Diagram of *IBM1* gene structure and its protein domains. The T-DNA insertion sites in *ibm1-4* and *ibm1-6* (red words) used in this study were labeled. Histograms showing the relative expression of IBM1 in flower buds of *ibm1-6* (B) and *ibm1-4* (C). Annealing sites for the primers is shown in (A). The error bar represents SD (standard deviation) of test. (D) Diagram showing *pIBM1*::*IBM1-FLAG* construction. (E) WT, *ibm1-6* and *pIBM1*::*IBM1-FLAG* complemented whole plants. (F) Western blots of protein extracted from flower buds of *ibm1-6* and the transgenic *pIBM1*::*IBM1-FLAG* complemented plants probed with an anti-FLAG antibody.
(TIF)

S2 Fig Centromere FISH analysis of meiosis in WT and *ibm1* meiocytes. FISH analysis with a 180 bp centromere repeat probe (red) of WT, *ibm1-4* and *ibm1-6* chromosomes spreads from pachytene (A), diakinesis (B), Metaphase I (C), and telophase II (D) meiocytes. Blue triangles on (A) show unsynapsed regions. The yellow arrows on (D) show chromosome fragments. Scale bar: 5 μm.
(TIF)

S3 Fig 5S rDNA FISH of WT and *ibm1* meiocytes. FISH analysis with 5S rDNA probe (red) of WT, *ibm1-4* and *ibm1-6* chromosome spreads from metaphase I meiocytes. The yellow arrows indicate nonhomologous chromosome interactions in *ibm1-4* and *ibm1-6*. Scale bar: 5 μm.
(TIF)

S4 Fig Immunostaining analysis of synaptonemal complex elements. (A) Immunofluorescence of ZYP1 (green) and SYN1 (magenta) in WT, *ibm1-4* and *ibm1-6* mutants at zygotene. (B) Immunofluorescence of ASY1 (red) in WT, *ibm1-4* and *ibm1-6* mutants at pachytene. Scale bar: 5 μm.
(TIF)

S5 Fig Recombination rate changed differently at different loci in *ibm1* mutant. (A) Location of fluorescent marker transgenes flanking intervals I2a, I2b, I3c, I5c and I5d on *Arabidopsis* chromosomes 2, 3 and 5. (B) Histogram showing genetic distances of I2a, I2b, I3c, I5c and I5d in WT and *ibm1-6*. * represents p-value<0.05, ** represents p-value<0.01 with two-tailed student *t* test.
(TIF)

S6 Fig IBM1 has *in vivo* H3K9me2 demethylase activity. (A) Transiently expressed *IBM1-GFP* in *N*. *benthamiana* is able to demethylate H3K9me2, but not H3K27me1, H3K4me1 and H3K4me3. Arrows indicate the nuclei with *IBM1* expression. (B) Quantitative analysis of relative integrated intensity of H3K9me2, H3K27me1, H3K4me1 and H3K4me3 in 30 pairs of nuclei for each histone modification. ** represents p-value< 0.01 with two-tailed student *t* test. The error bar represents SD of each group. Scale bar: 20 μm.
(TIF)

S7 Fig Immunostaining analysis of H3K9me2 and H3K4me3. Immunofluorescence of H3K9me2 (red) in meiocytes of WT, *ibm1-4* and *ibm1-6* mutants at zygotene in (A), and pachytene in (B). (C) Quantification of integrated intensity of H3K9me2 from (A) and (B). The signals of H3K4me3 (red) in meiocytes of WT, *ibm1-4* and *ibm1-6* mutants at zygotene in (D), and pachytene in (E). (F) Quantification of integrated intensity of H3K4me3 from (D) and (E). * p-value< 0.05, ** p-value< 0.01 with two-tailed student *t* test, n.s: Not significant. The error bars represent the SD of each group. Scale bar: 5 μm.
(TIF)

S8 Fig *suvh4*/*kyp* and *cmt3* suppress the fertility and meiotic defects in *ibm1*. (A) Alexander staining of anthers from WT, *ibm1-4*, *ibm1-6*, *suvh4*, *cmt3*, *ibm1-4 cmt3*, *ibm1-4 suvh4*, *ibm1-6 cmt3* and *ibm1-6 suvh4* plants. Scale bar: 50 μm. (B) Histogram showing percentage of cells with asynapsis (red) or complete synapsis (blue) in Metaphase I. (C) Histogram showing percentage of cells with univalent (blue) or without univalent (orange) in Metaphase I.
(TIF)

S9 Fig Immunostaining analysis of 5mC level in combination with centromere FISH. Immunofluorescence with and antibody against 5mC (green) coupled with with FISH using a 180 bp centromere probe (magenta) in meiocytes of WT, *ibm1-4* and *ibm1-6* mutants at zygotene in (A), and pachytene in (B). (C) Quantification of integrated intensity of 5mC from (A) and (B). * p-value< 0.05, ** p-value< 0.01 with two-tailed student *t* test, n.s: not significant. The error bars represent the SD of each group. Scale bar: 5 μm.
(TIF)

S10 Fig The effect of IBM1 on the change of CHG methylation level of TEs. (A) Heat map showing methylation distribution of CHG methylation in TEs with reduced CHG methylation. (B) CHG methylation changes of different types of TEs in *ibm1-6* and *ibm1-6 cmt3-7*.
(TIF)

S11 Fig CHG methylation of TEs decrease at different level based on their distance from genes. CHG methylation changes of different types of TEs in *ibm1-6* and *ibm1-6 cmt3-7* based on their positions. These TEs are classified three categories: intragenic TEs, out of genes and <2kb from genes (<2kb TEs for short), out of genes and >2kb from genes (>2kb TEs for short)
(TIF)

S12 Fig Comparison of differentially expressed genes between meiocytes WT and *ibm1* mutant under different cutoff of RPKM values. (A) MA plot of all genes detected in transcriptromes of meiocytes WT and *ibm1* mutant. Differentially expressed genes (DEGs) identified by using DESeq2 (fold change ≥2 and p-value <0.01). DEGs exhibiting RPKM values >2 are colored in blue and those with RPKM≤2 in red. (B) Illustration of RPKM values in meiocytes WT and *ibm1* mutant (C) Illustraion of number of DEGs using different RPKM cutoffs (from 1 to 10).
(TIF)

S13 Fig Biological process enrichment analysis. (A) Gene ontology analysis of biological processes in leaf-specific downregulated genes (leDRGs), meiocyte-specific downregulated genes (meDRGs), leaf-specific upregulated genes (leURGs), meiocyte upregulated genes (meURGs). (B) Gene ontology analysis of biological processes in genes with restored expression in *ibm1-6 cmt3-7*. (C) Gene ontology analysis of biological processes in genes with restored expression in *ibm1-6 cmt3-7* corresponding to significant elevation of gene body CHG methylation in *ibm1*.
(TIF)

S14 Fig CHG methylation and gene expression in meiotic known genes and 437 genes with restored expression and elevated CHG methylation. (A) Illustration of distribution of CHG methylation on 128 meiosis related genes for WT, *ibm1* single mutant and *ibm1 cmt3* double mutant. (B) Scatter diagram shows Log2 (fold change) gene expression on 128 meiosis related genes of *ibm1* mutant compared with WT for meiocytes and leaves, respectively. (C) Gene expression changes in *ibm1* single mutant and *ibm1 cmt3* double mutant compared with WT for meiocytes. (D) Illustration of distribution of CHG methylation for 437 selected genes. (E) Comparison of genes expression changes in *ibm1* single mutant compared with WT and (F) in *ibm1* single mutant and *ibm1 cmt3* double mutant compared with WT for 437 selected genes.
(TIF)

S15 Fig DNA methylation and expression level of *AML3*, *AML4* and *AML5* in 3-week old leaves. (A) Distribution of CG (top rows), CHG (middle rows) and CHH (bottom rows) methylation within gene body of *AML3* (top row), *AML4* (middle row) and *AML5* (bottom row). Red rectangles indicate elevation of gene body CHG methylation in *AML3-5*. (B) CHOP-PCR analysis of *AML3-5* in WT, *ibm1-4*, *ibm1-6*, *cmt3*, *suvh4*, *ibm1-4 cmt3*, *ibm1-4 suvh4*, *ibm1-6 cmt3* and *ibm1-6 suvh4*. (C) Relative expression of *AML3-5* in leaves of WT and *ibm1-4*. Relative expression is indicated by FPKM fold change by DEseq2 analysis. Two biological replicates were used. n.s represents not significant, ** represents p-value<0.05.
(TIF)

S16 Fig Ectopic expression of *AML5* partially restored fertility and meiosis of *ibm1* mutant. (A) Quantification of seeds per silique from WT, *ibm1-6* and 20 independent lines. 5 siliques per plant were used for quantification. (B) Quantification of viable pollen grains per anther from WT, *ibm1-6* plants and 20 independent lines. 10 anthers per plant were used for quantification. The data of (A) and (B) are shown as mean ± SD. In (A) and (B), 20 independent lines were respectively compared to *ibm1-6* mutant with two-tailed student *t* test. n.s stands for not significant, * P-value<0.05, ** P-value<0.01, *** P-value<0.001. WT, *ibm1-6* plants and 4 independent lines (#1, #2, #19 and #20) of *pAct7*::*AML5/ibm1-6*. (C) Anthers with alexander staining from WT, *ibm1-6* and two independent lines (#19 and #20) of *pAct7*::*AML5/ibm1-6*. (D) Chromosome spreads of pachytene and metaphase I from WT, *ibm1-6* and two independent lines (#19 and #20) of *pAct7*::*AML5/ibm1-6* meiocytes. (E) Western blots of protein extracted from flower buds of *ibm1-6* and two transgenic *pAct7*::*AML5/ibm1-6* plants (#19 and #20) probed with an anti-FLAG antibody. Scale bars: (C): 50 μm, (D): 5 μm.
(TIF)

S1 Table Quantification of silique lengths and seed numbers. Statistical data were taken by comparing WT×WT with each of *ibm-4*×*ibm-4*, WT×*ibm1-4*, *ibm1-4*×WT, *ibm-6*×*ibm-6*, WT×*ibm1-6* and *ibm1-6*×WT. * represents p-value<0.05, ** represents p-value<0.01, with two-tailed student *t* test.
(PDF)

S2 Table Chiasmata frequency per chromosome in WT, *ibm1-4* and *ibm1-6*. Statistical data were taken by comparing WT with *ibm-4* or *ibm1-6*. * represents p-value<0.05, ** represents p-value<0.01, with two-tailed student *t* test.
(PDF)

S3 Table Expression of different meiotic genes in different genotype. (PDF)

S4 Table Fertility analysis of 20 *AML5*/*ibm1-6* plants. Five siliques were analyzed in each plant. Statistical data were taken by comparing each *AML5*/*ibm1-6 plant* with *ibm-6*. Number of plants (#1-#2) were ranked by normal seed numbers. n.s represents not significant, * represents p-value<0.05, ** represents p-value<0.01, *** represents p-value<0.001 with two-tailed student *t* test.
(PDF)

S5 Table Primers used in this study. (PDF)
