# Supplementary figures and images for: Histone demethylase IBM1-mediated meiocyte gene expression ensures meiotic chromosome synapsis and recombination
Source: PLoS Genet. 2022 Feb 22;18(2):e1010041. doi: 10.1371/journal.pgen.1010041 (PMC8896719; doi:10.1371/journal.pgen.1010041)

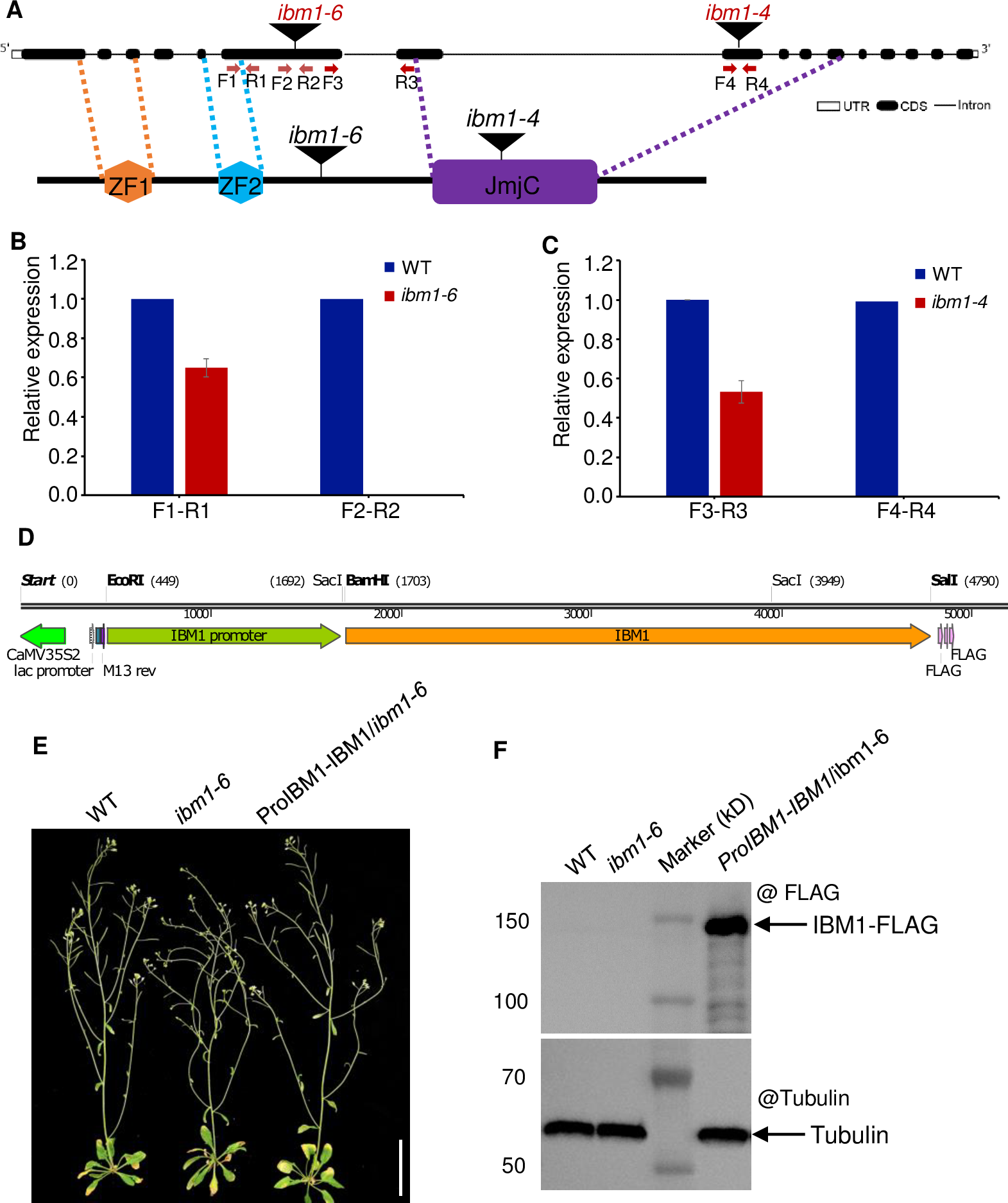

Supplement: S1 Fig — (A) Diagram of IBM1 gene structure and its protein domains. The T-DNA insertion sites in ibm1-4 and ibm1-6 (red words) used in this study were labeled. Histograms showing the relative expression of IBM1 in flower buds of ibm1-6 (B) and ibm1-4 (C). Annealing sites for the primers is shown in (A). The error bar represents SD (standard deviation) of test. (D) Diagram showing pIBM1::IBM1-FLAG construction. (E) WT, ibm1-6 and pIBM1::IBM1-FLAG complemented whole plants. (F) Western blots of protein extracted from flower buds of ibm1-6 and the transgenic pIBM1::IBM1-FLAG complemented plants probed with an anti-FLAG antibody. (TIF) [file pgen.1010041.s001.tif]

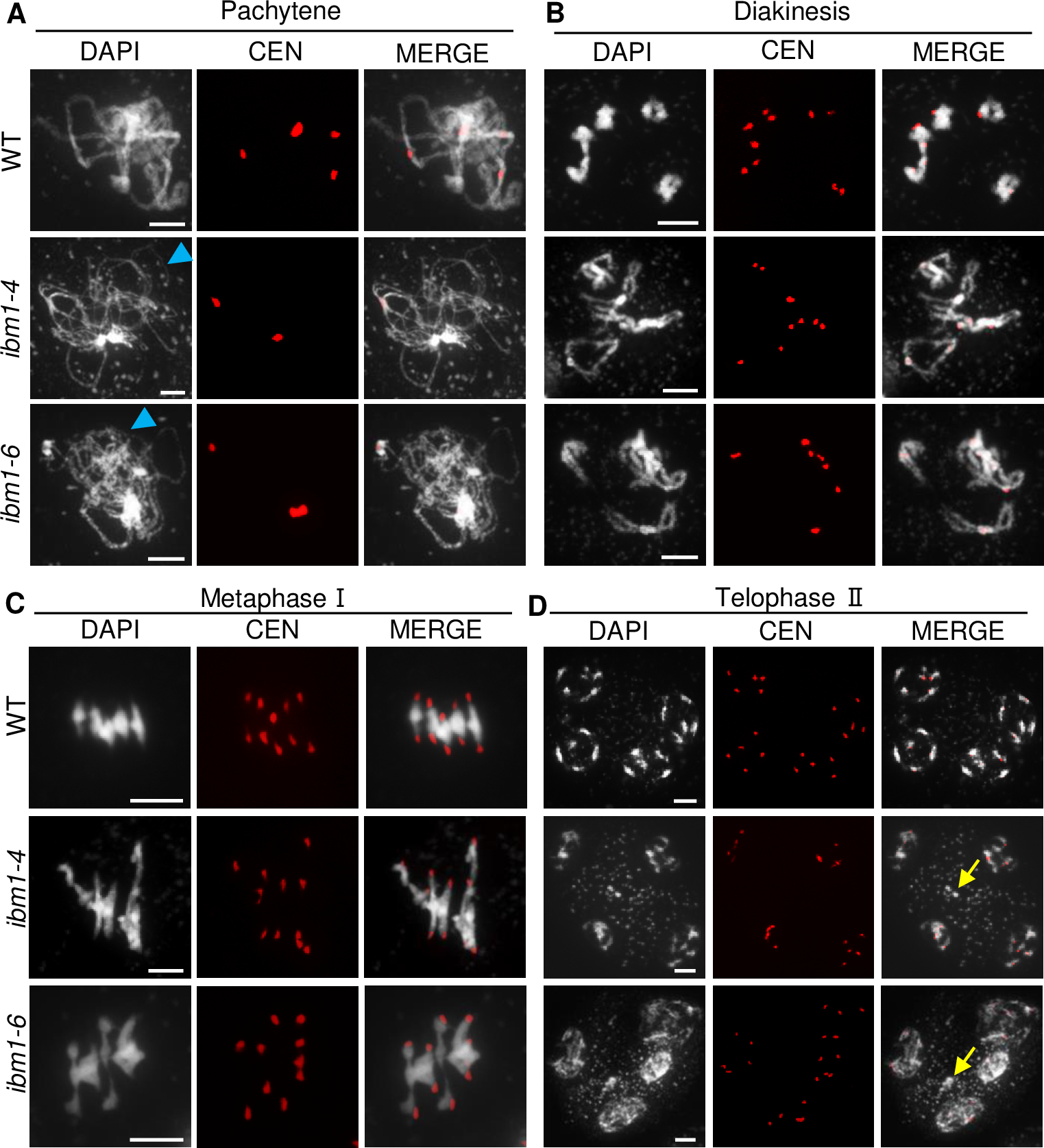

Supplement: S2 Fig — FISH analysis with a 180 bp centromere repeat probe (red) of WT, ibm1-4 and ibm1-6 chromosomes spreads from pachytene (A), diakinesis (B), Metaphase I (C), and telophase II (D) meiocytes. Blue triangles on (A) show unsynapsed regions. The yellow arrows on (D) show chromosome fragments. Scale bar: 5 μm. (TIF) [file pgen.1010041.s002.tif]

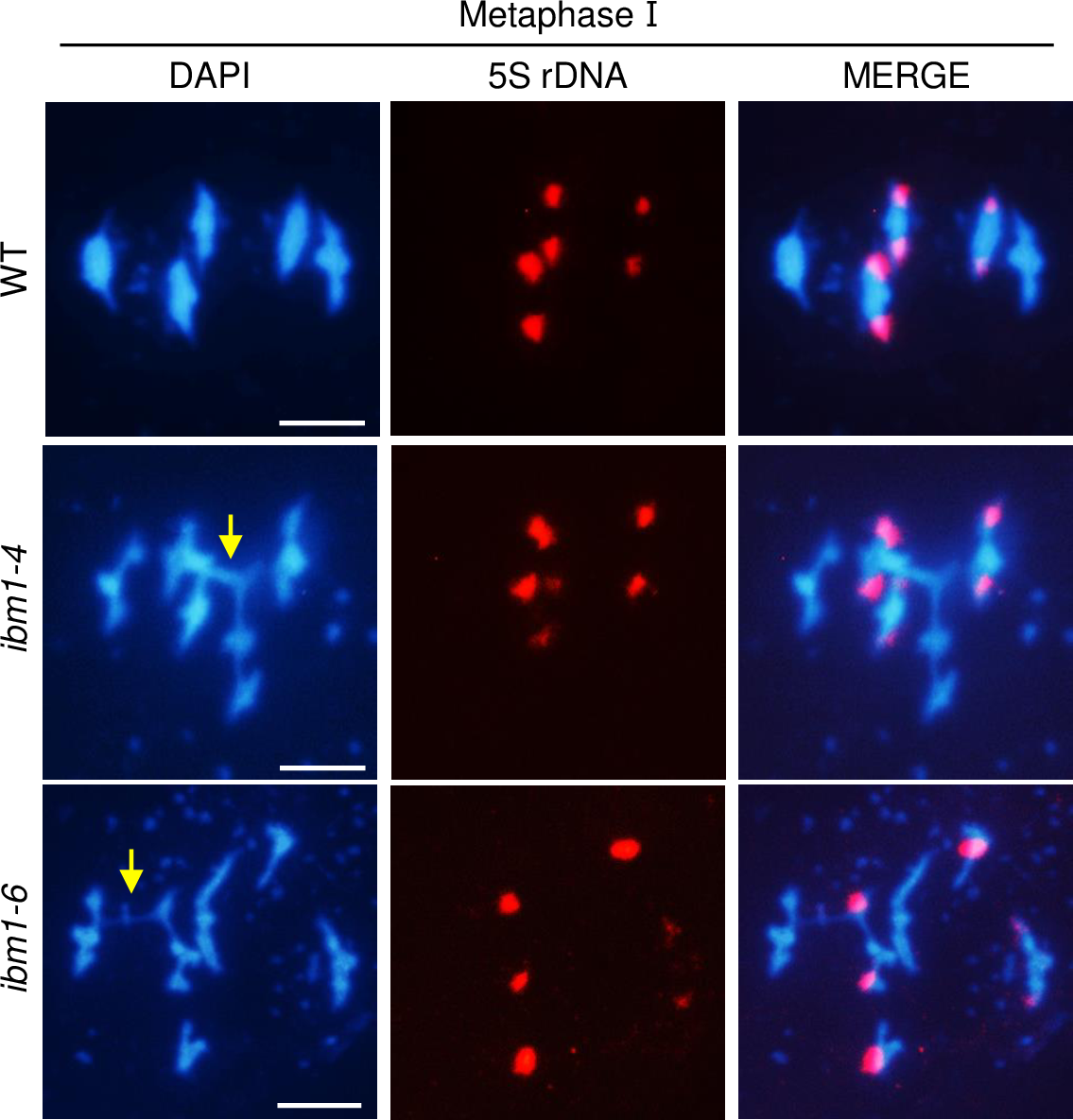

Supplement: S3 Fig — FISH analysis with 5S rDNA probe (red) of WT, ibm1-4 and ibm1-6 chromosome spreads from metaphase I meiocytes. The yellow arrows indicate nonhomologous chromosome interactions in ibm1-4 and ibm1-6. Scale bar: 5 μm. (TIF) [file pgen.1010041.s003.tif]

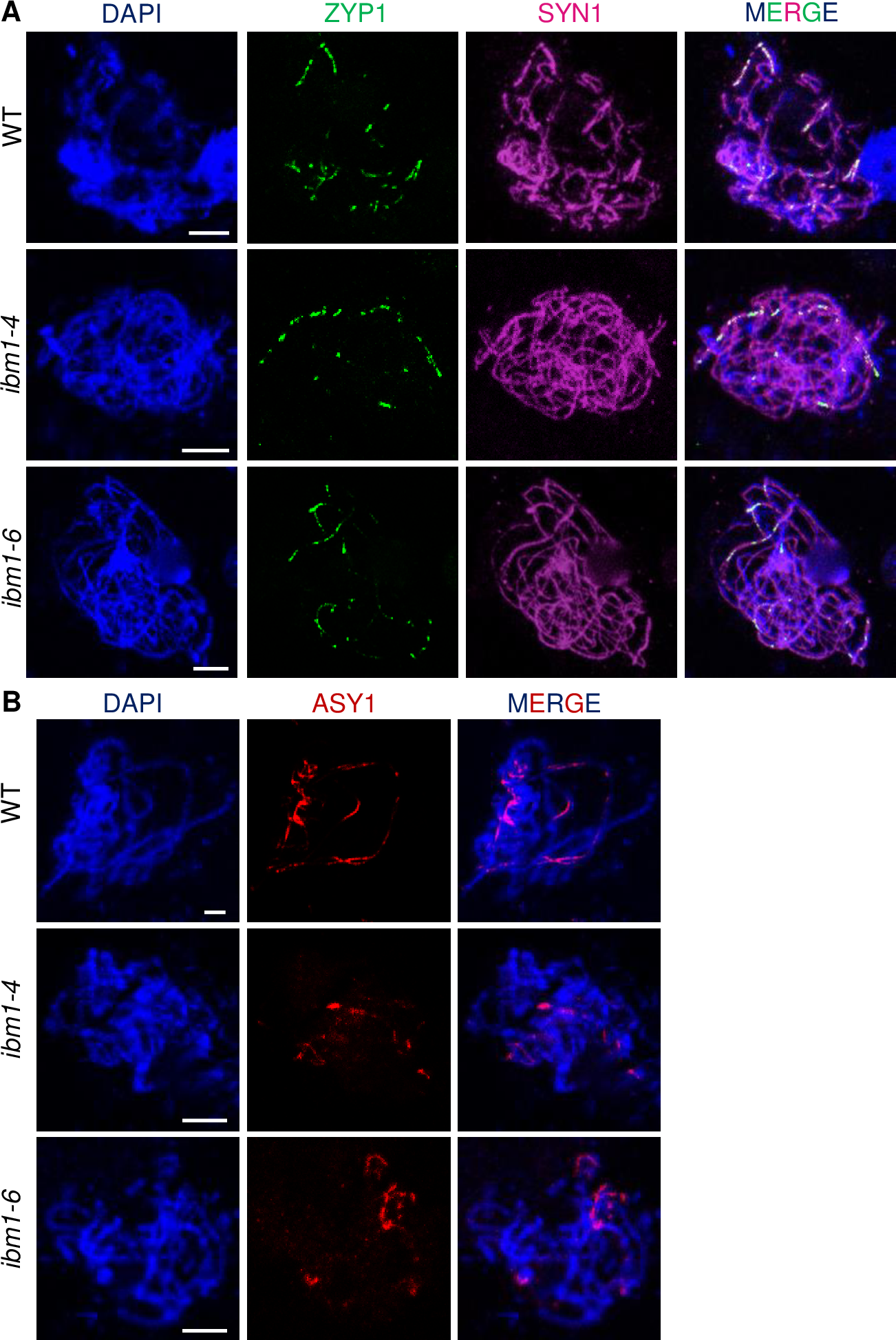

Supplement: S4 Fig — (A) Immunofluorescence of ZYP1 (green) and SYN1 (magenta) in WT, ibm1-4 and ibm1-6 mutants at zygotene. (B) Immunofluorescence of ASY1 (red) in WT, ibm1-4 and ibm1-6 mutants at pachytene. Scale bar: 5 μm. (TIF) [file pgen.1010041.s004.tif]

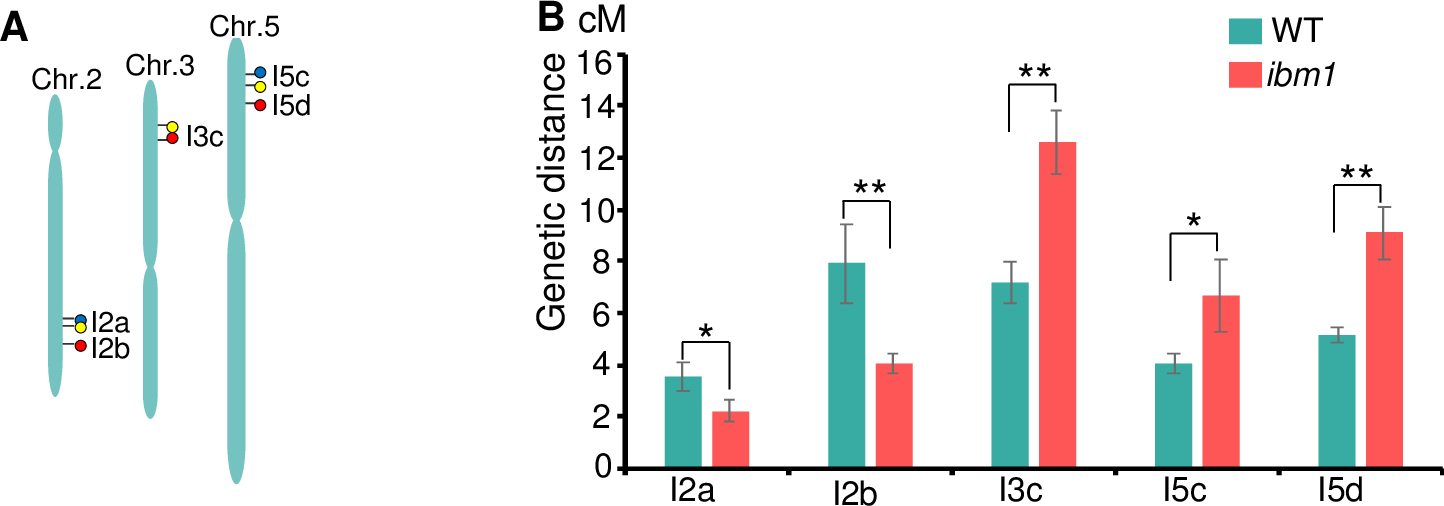

Supplement: S5 Fig — (A) Location of fluorescent marker transgenes flanking intervals I2a, I2b, I3c, I5c and I5d on Arabidopsis chromosomes 2, 3 and 5. (B) Histogram showing genetic distances of I2a, I2b, I3c, I5c and I5d in WT and ibm1-6. * represents p-value<0.05, ** represents p-value<0.01 with two-tailed student t test. (TIF) [file pgen.1010041.s005.tif]

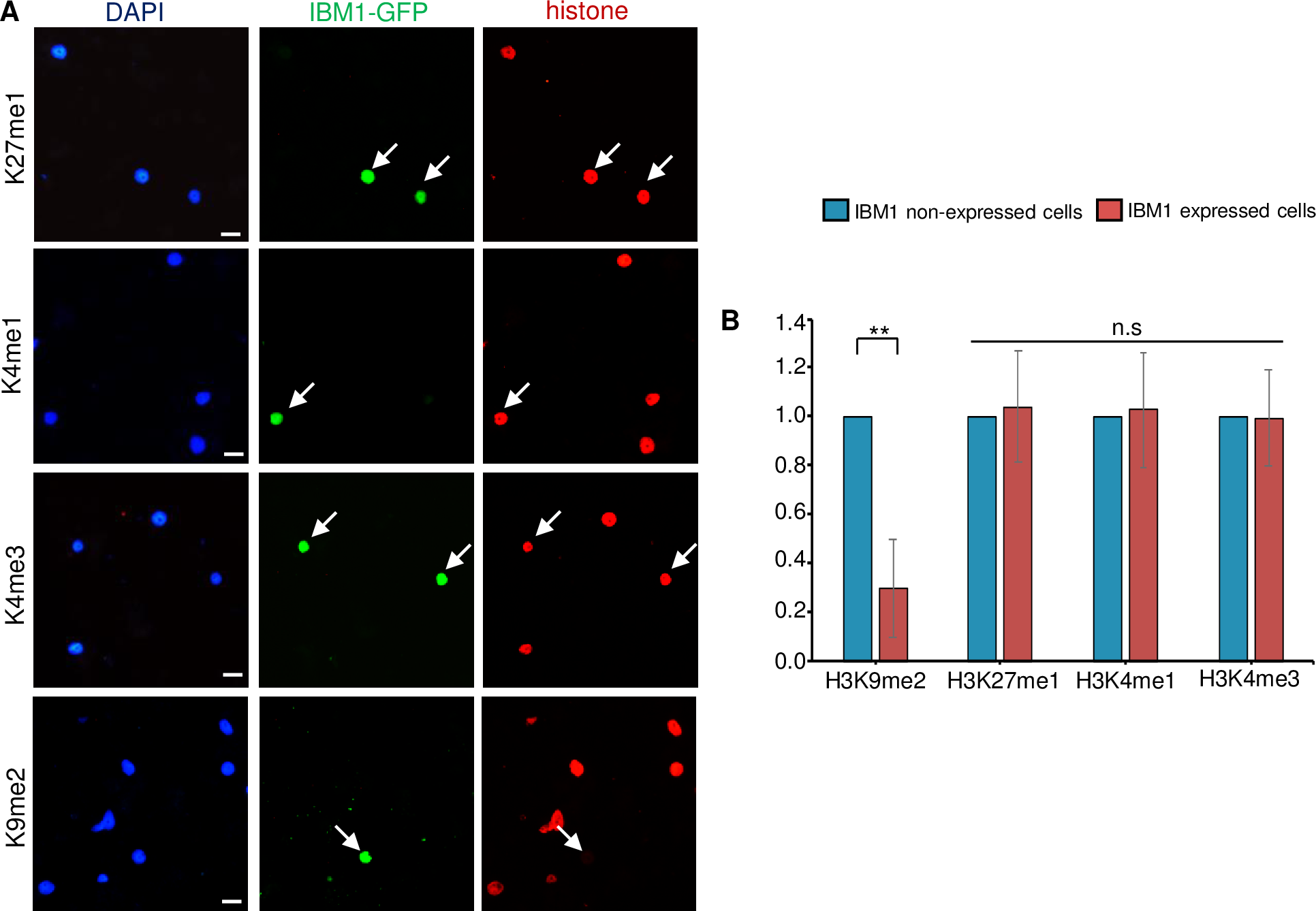

Supplement: S6 Fig — (A) Transiently expressed IBM1-GFP in N. benthamiana is able to demethylate H3K9me2, but not H3K27me1, H3K4me1 and H3K4me3. Arrows indicate the nuclei with IBM1 expression. (B) Quantitative analysis of relative integrated intensity of H3K9me2, H3K27me1, H3K4me1 and H3K4me3 in 30 pairs of nuclei for each histone modification. ** represents p-value< 0.01 with two-tailed student t test. The error bar represents SD of each group. Scale bar: 20 μm. (TIF) [file pgen.1010041.s006.tif]

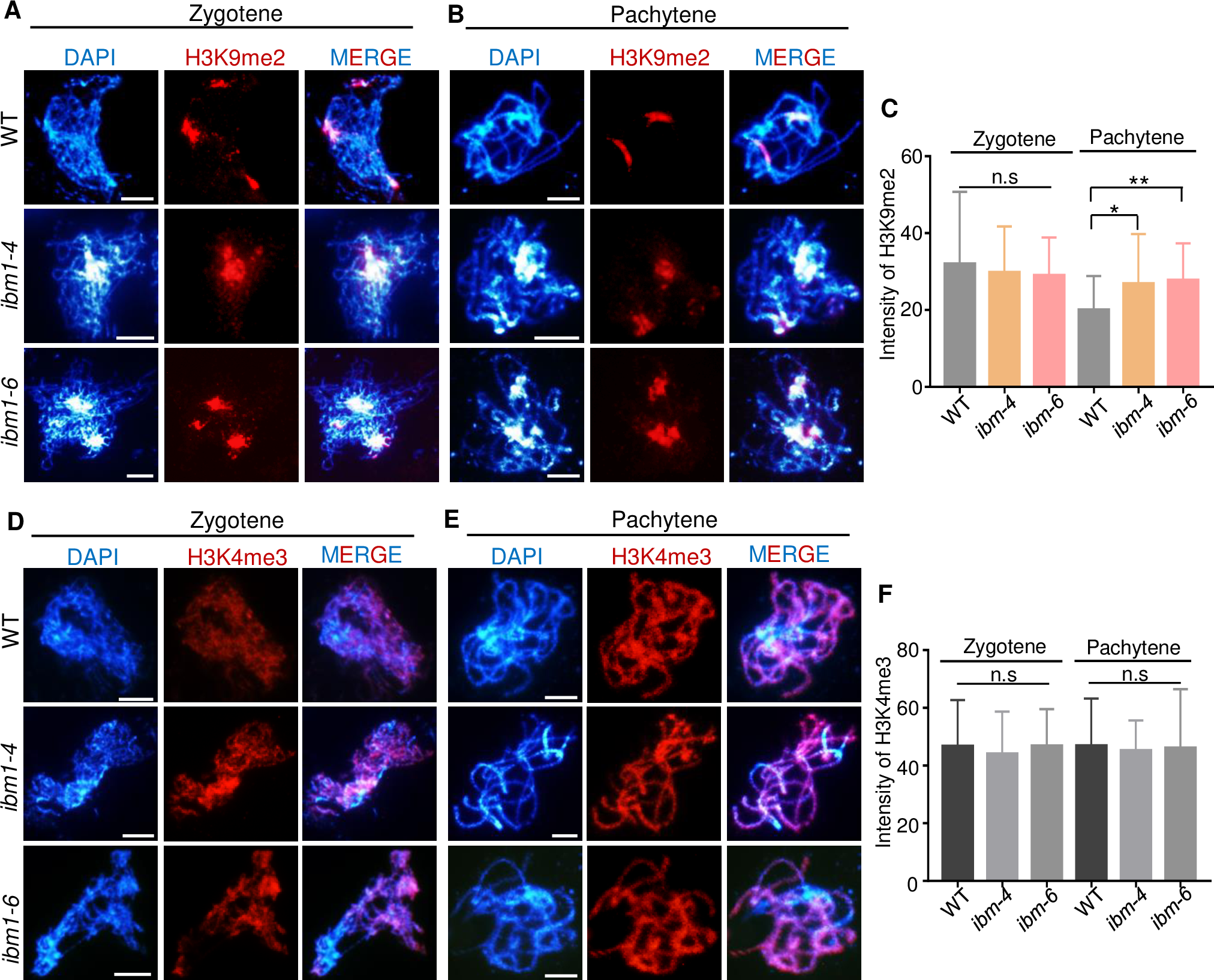

Supplement: S7 Fig — Immunofluorescence of H3K9me2 (red) in meiocytes of WT, ibm1-4 and ibm1-6 mutants at zygotene in (A), and pachytene in (B). (C) Quantification of integrated intensity of H3K9me2 from (A) and (B). The signals of H3K4me3 (red) in meiocytes of WT, ibm1-4 and ibm1-6 mutants at zygotene in (D), and pachytene in (E). (F) Quantification of integrated intensity of H3K4me3 from (D) and (E). * p-value< 0.05, ** p-value< 0.01 with two-tailed student t test, n.s: Not significant. The error bars represent the SD of each group. Scale bar: 5 μm. (TIF) [file pgen.1010041.s007.tif]

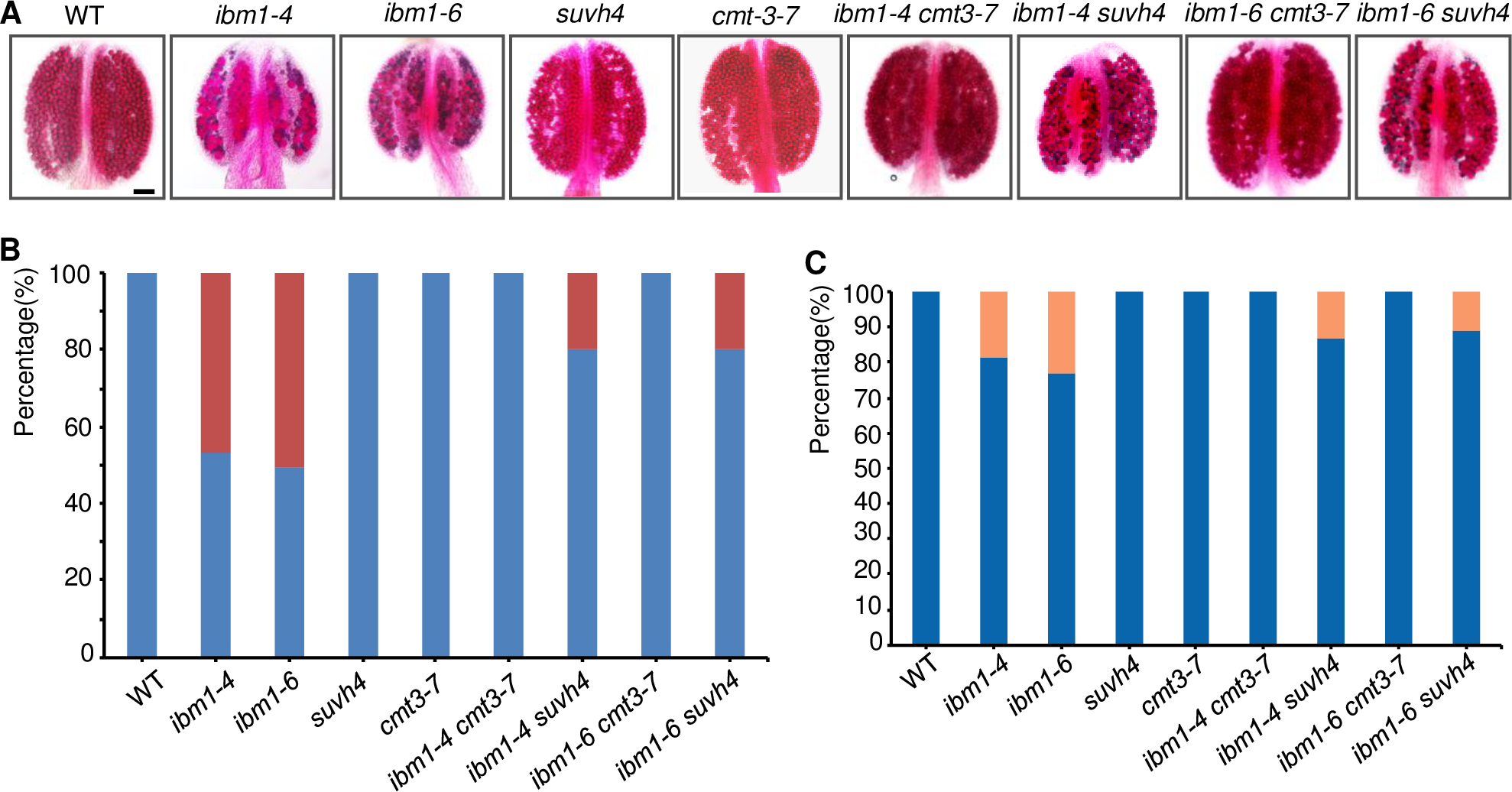

Supplement: S8 Fig — (A) Alexander staining of anthers from WT, ibm1-4, ibm1-6, suvh4, cmt3, ibm1-4 cmt3, ibm1-4 suvh4, ibm1-6 cmt3 and ibm1-6 suvh4 plants. Scale bar: 50 μm. (B) Histogram showing percentage of cells with asynapsis (red) or complete synapsis (blue) in Metaphase I. (C) Histogram showing percentage of cells with univalent (blue) or without univalent (orange) in Metaphase I. (TIF) [file pgen.1010041.s008.tif]

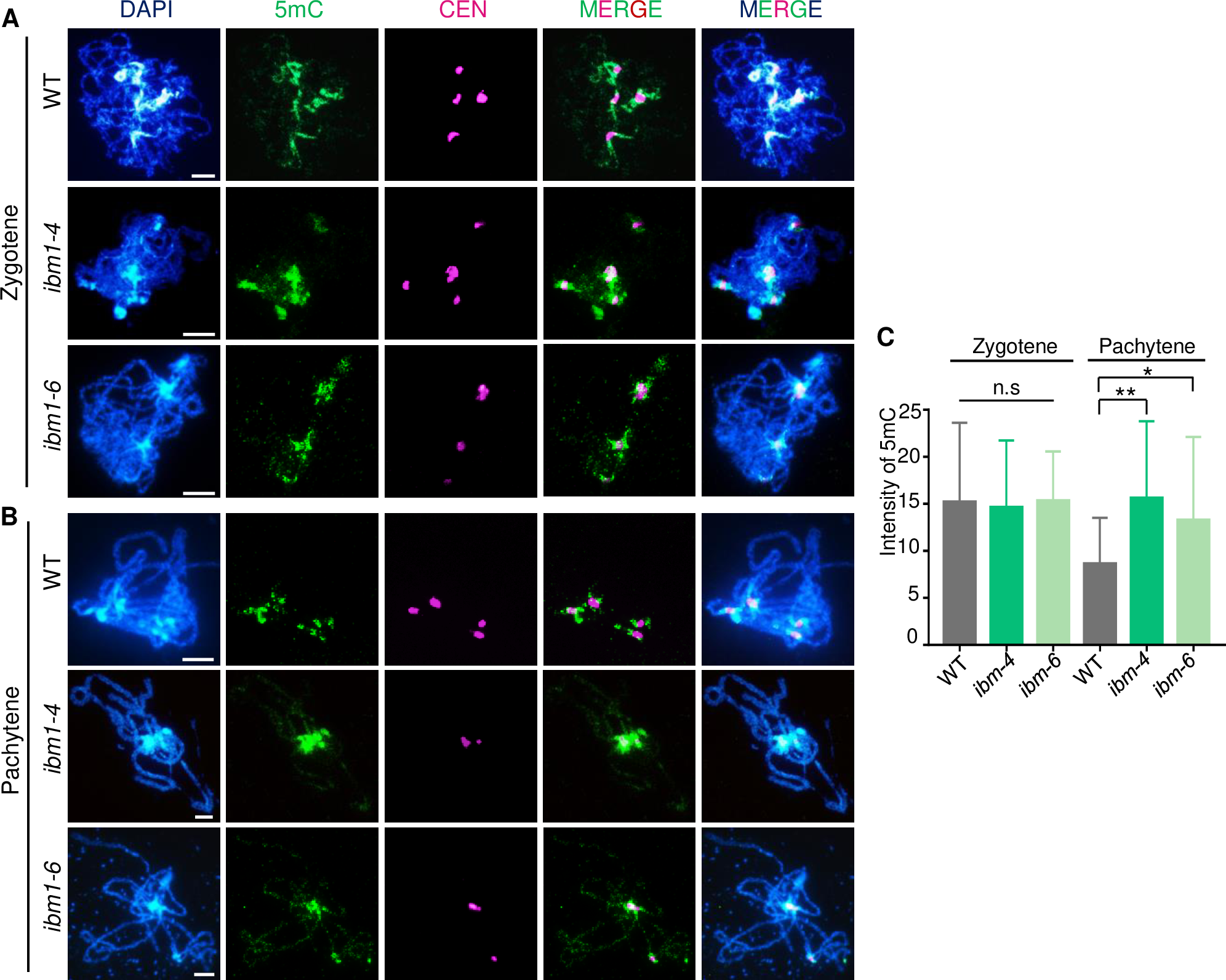

Supplement: S9 Fig — Immunofluorescence with and antibody against 5mC (green) coupled with with FISH using a 180 bp centromere probe (magenta) in meiocytes of WT, ibm1-4 and ibm1-6 mutants at zygotene in (A), and pachytene in (B). (C) Quantification of integrated intensity of 5mC from (A) and (B). * p-value< 0.05, ** p-value< 0.01 with two-tailed student t test, n.s: not significant. The error bars represent the SD of each group. Scale bar: 5 μm. (TIF) [file pgen.1010041.s009.tif]

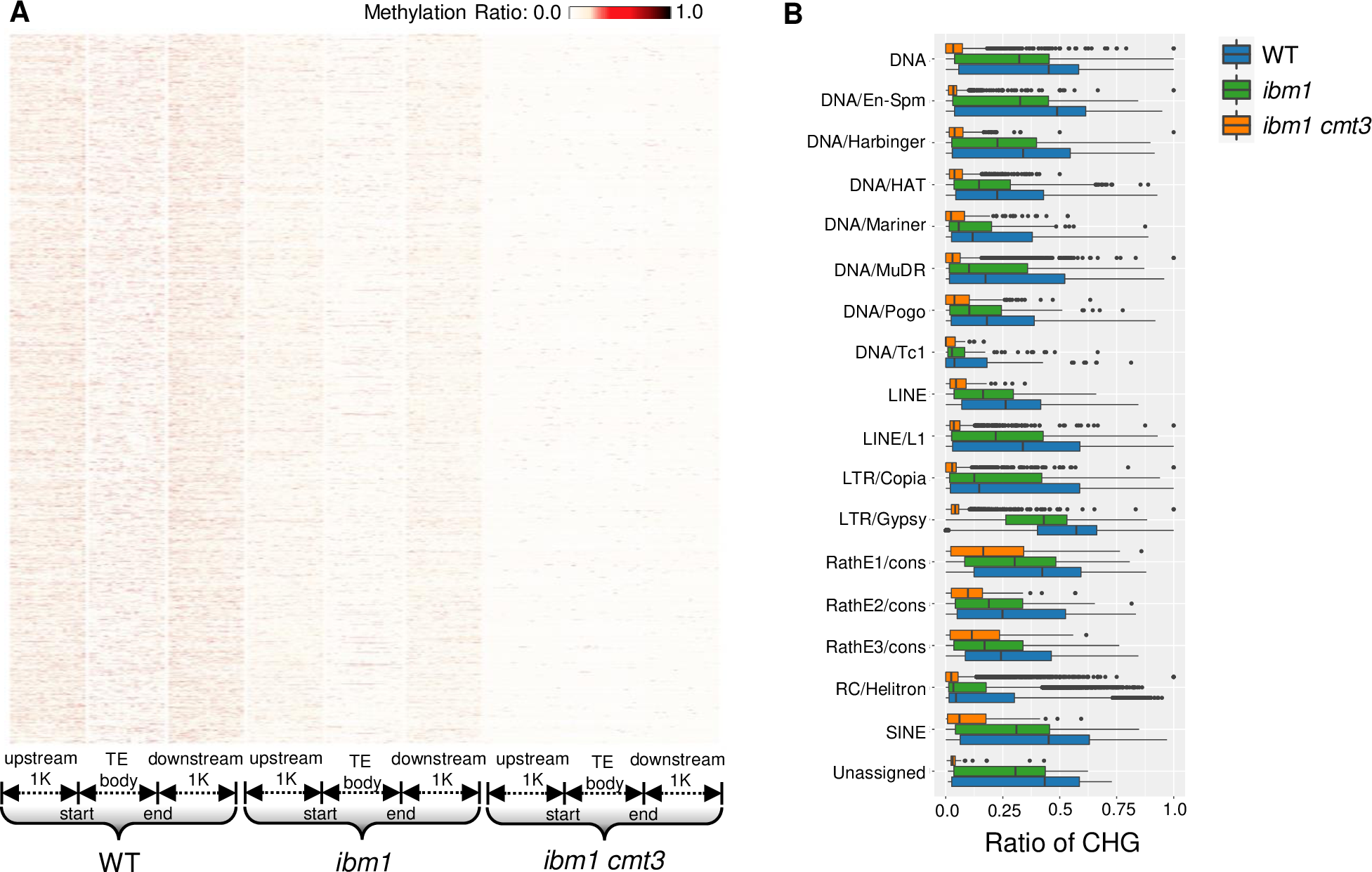

Supplement: S10 Fig — (A) Heat map showing methylation distribution of CHG methylation in TEs with reduced CHG methylation. (B) CHG methylation changes of different types of TEs in ibm1-6 and ibm1-6 cmt3-7. (TIF) [file pgen.1010041.s010.tif]

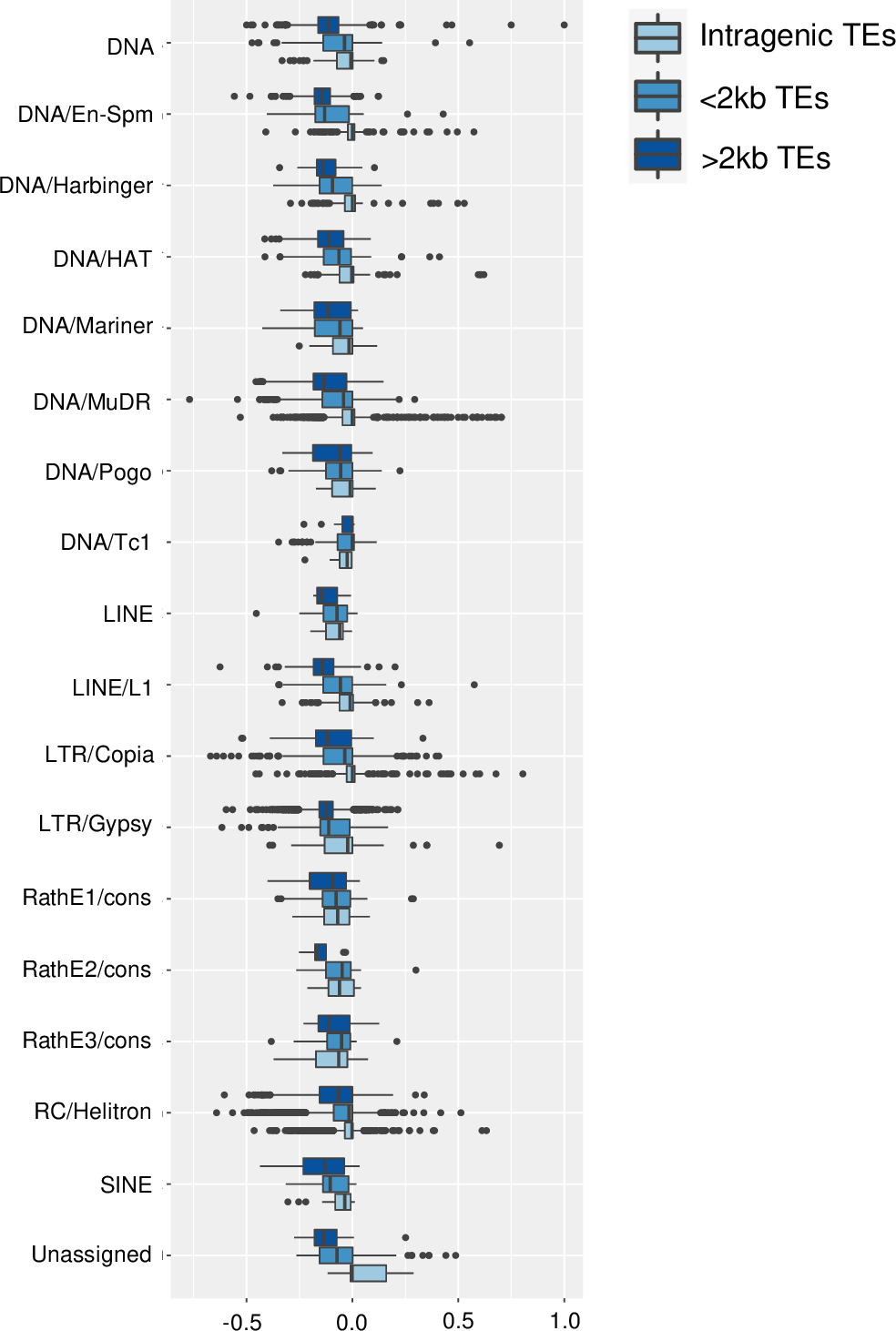

Supplement: S11 Fig — CHG methylation changes of different types of TEs in ibm1-6 and ibm1-6 cmt3-7 based on their positions. These TEs are classified three categories: intragenic TEs, out of genes and <2kb from genes (<2kb TEs for short), out of genes and >2kb from genes (>2kb TEs for short) (TIF) [file pgen.1010041.s011.tif]

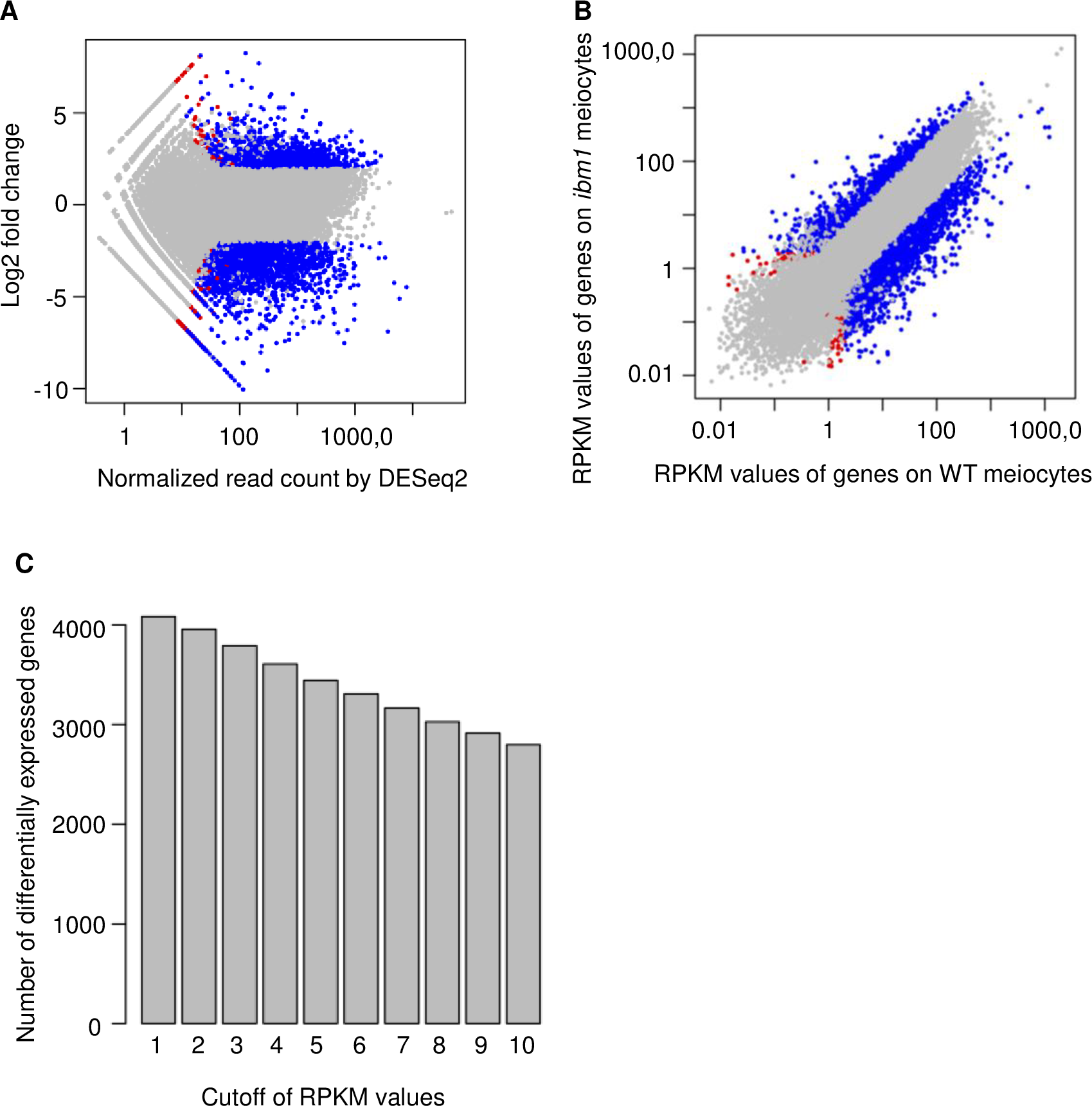

Supplement: S12 Fig — (A) MA plot of all genes detected in transcriptromes of meiocytes WT and ibm1 mutant. Differentially expressed genes (DEGs) identified by using DESeq2 (fold change ≥2 and p-value <0.01). DEGs exhibiting RPKM values >2 are colored in blue and those with RPKM≤2 in red. (B) Illustration of RPKM values in meiocytes WT and ibm1 mutant (C) Illustraion of number of DEGs using different RPKM cutoffs (from 1 to 10). (TIF) [file pgen.1010041.s012.tif]

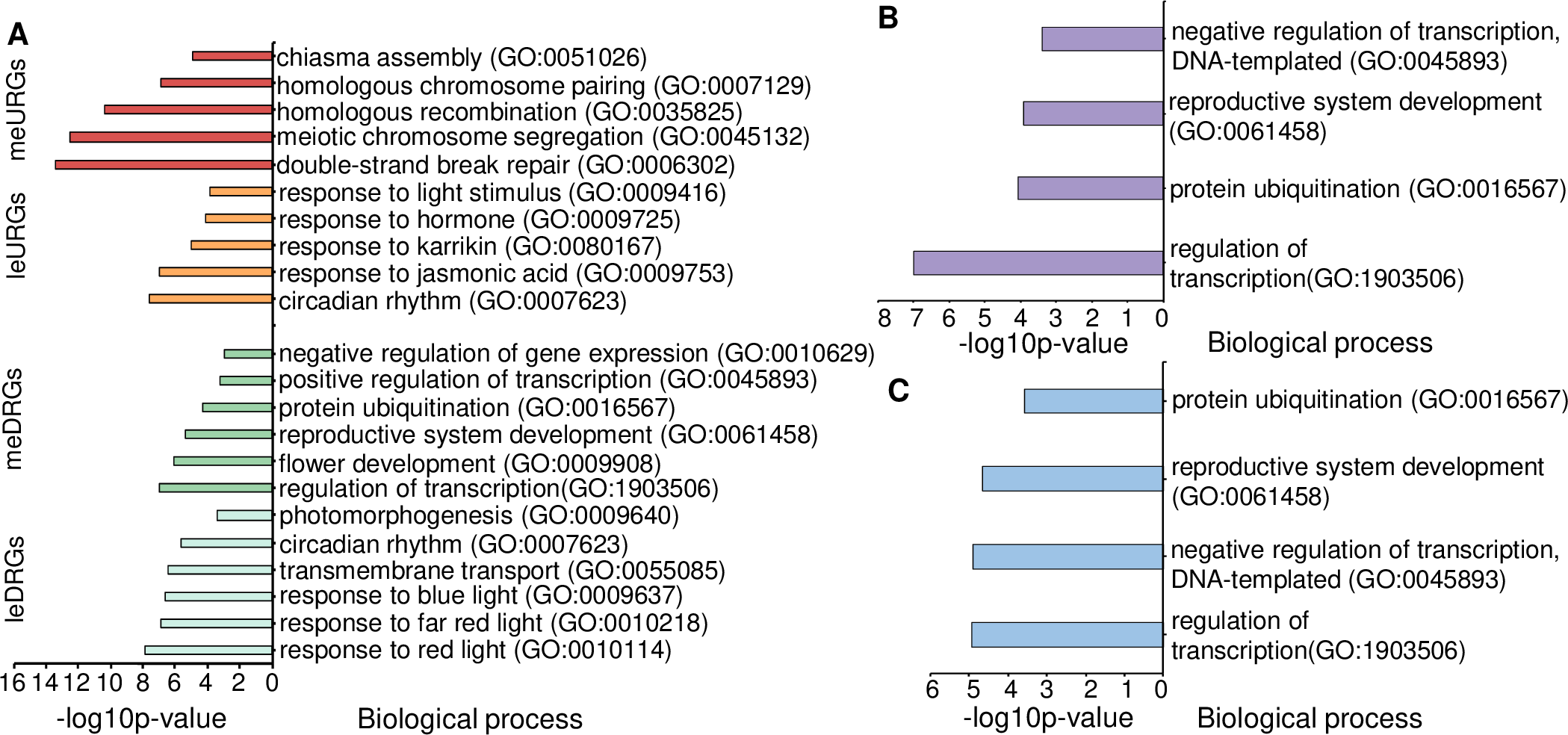

Supplement: S13 Fig — (A) Gene ontology analysis of biological processes in leaf-specific downregulated genes (leDRGs), meiocyte-specific downregulated genes (meDRGs), leaf-specific upregulated genes (leURGs), meiocyte upregulated genes (meURGs). (B) Gene ontology analysis of biological processes in genes with restored expression in ibm1-6 cmt3-7. (C) Gene ontology analysis of biological processes in genes with restored expression in ibm1-6 cmt3-7 corresponding to significant elevation of gene body CHG methylation in ibm1. (TIF) [file pgen.1010041.s013.tif]

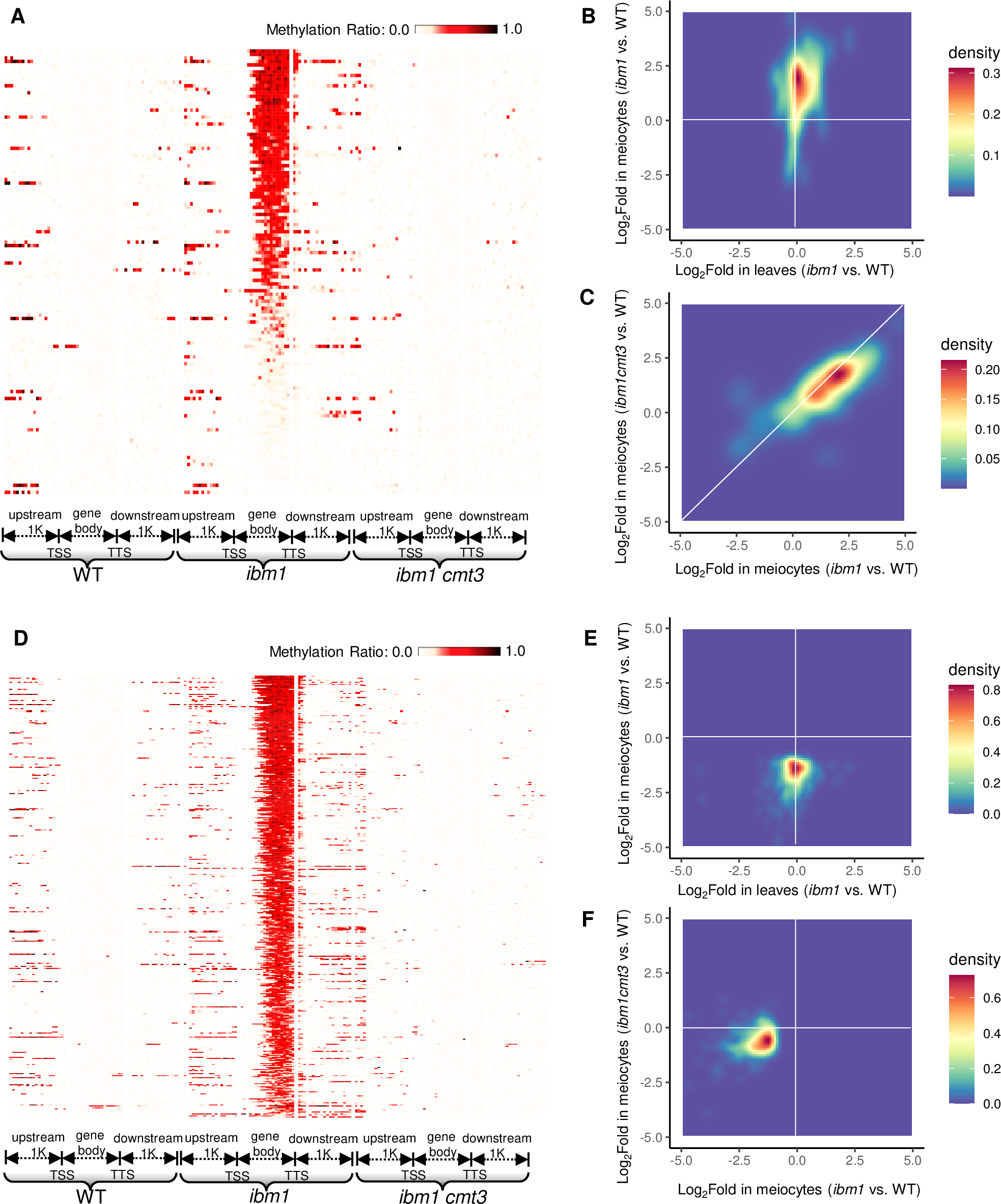

Supplement: S14 Fig — (A) Illustration of distribution of CHG methylation on 128 meiosis related genes for WT, ibm1 single mutant and ibm1 cmt3 double mutant. (B) Scatter diagram shows Log2 (fold change) gene expression on 128 meiosis related genes of ibm1 mutant compared with WT for meiocytes and leaves, respectively. (C) Gene expression changes in ibm1 single mutant and ibm1 cmt3 double mutant compared with WT for meiocytes. (D) Illustration of distribution of CHG methylation for 437 selected genes. (E) Comparison of genes expression changes in ibm1 single mutant compared with WT and (F) in ibm1 single mutant and ibm1 cmt3 double mutant compared with WT for 437 selected genes. (TIF) [file pgen.1010041.s014.tif]

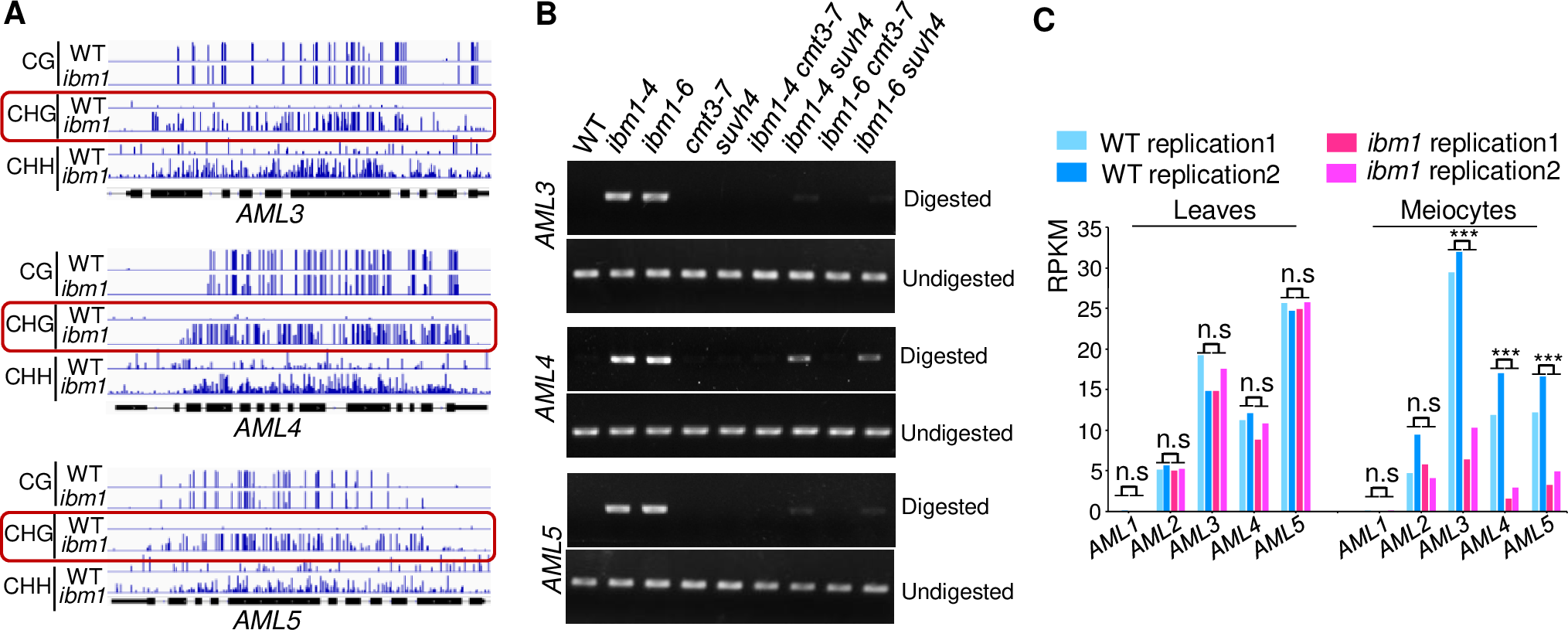

Supplement: S15 Fig — (A) Distribution of CG (top rows), CHG (middle rows) and CHH (bottom rows) methylation within gene body of AML3 (top row), AML4 (middle row) and AML5 (bottom row). Red rectangles indicate elevation of gene body CHG methylation in AML3-5. (B) CHOP-PCR analysis of AML3-5 in WT, ibm1-4, ibm1-6, cmt3, suvh4, ibm1-4 cmt3, ibm1-4 suvh4, ibm1-6 cmt3 and ibm1-6 suvh4. (C) Relative expression of AML3-5 in leaves of WT and ibm1-4. Relative expression is indicated by FPKM fold change by DEseq2 analysis. Two biological replicates were used. n.s represents not significant, ** represents p-value<0.05. (TIF) [file pgen.1010041.s015.tif]

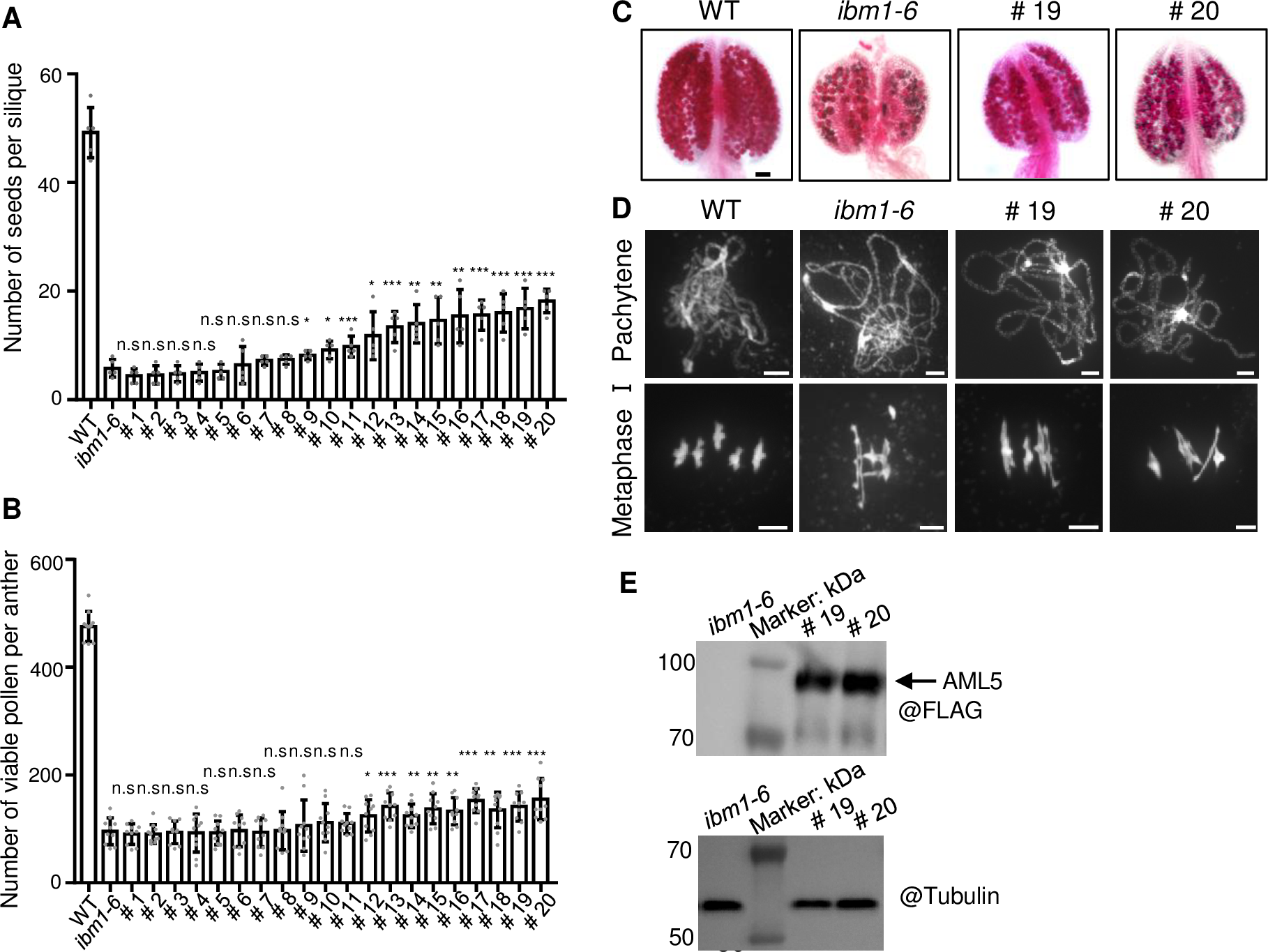

Supplement: S16 Fig — (A) Quantification of seeds per silique from WT, ibm1-6 and 20 independent lines. 5 siliques per plant were used for quantification. (B) Quantification of viable pollen grains per anther from WT, ibm1-6 plants and 20 independent lines. 10 anthers per plant were used for quantification. The data of (A) and (B) are shown as mean ± SD. In (A) and (B), 20 independent lines were respectively compared to ibm1-6 mutant with two-tailed student t test. n.s stands for not significant, * P-value<0.05, ** P-value<0.01, *** P-value<0.001. WT, ibm1-6 plants and 4 independent lines (#1, #2, #19 and #20) of pAct7::AML5/ibm1-6. (C) Anthers with alexander staining from WT, ibm1-6 and two independent lines (#19 and #20) of pAct7::AML5/ibm1-6. (D) Chromosome spreads of pachytene and metaphase I from WT, ibm1-6 and two independent lines (#19 and #20) of pAct7::AML5/ibm1-6 meiocytes. (E) Western blots of protein extracted from flower buds of ibm1-6 and two transgenic pAct7::AML5/ibm1-6 plants (#19 and #20) probed with an anti-FLAG antibody. Scale bars: (C): 50 μm, (D): 5 μm. (TIF) [file pgen.1010041.s016.tif]
