# Supplementary material for: Histone demethylase IBM1-mediated meiocyte gene expression ensures meiotic chromosome synapsis and recombination
Source: PLoS Genet. 2022 Feb 22;18(2):e1010041. doi: 10.1371/journal.pgen.1010041 (PMC8896719; doi:10.1371/journal.pgen.1010041)
Supplement: S1 Table — Statistical data were taken by comparing WT×WT with each of ibm-4×ibm-4, WT×ibm1-4, ibm1-4×WT, ibm-6×ibm-6, WT×ibm1-6 and ibm1-6×WT. * represents p-value<0.05, ** represents p-value<0.01, with two-tailed student t test. (PDF) [file pgen.1010041.s017.pdf]

S1 Table. Quantification of silique lengths and seed numbers.

♀ × ♂	Silique length (mm)	Normal seeds number	Aborted seeds number
WT× WT (n=21)	14.5±0.7	48.0±3.0	0.2±0.4
ibm-4×*ibm-4* (n=22)	3.6±1.5**	2.6±2.4**	0.7±1.2$^{n.s}$
WT×*ibm1-4* (n=23)	5.0±0.9**	7.7±3.8**	0.3±0.4$^{n.s}$
ibm1-4×WT (n=20)	10.2±2.1**	14.3±5.2**	1.8±2.2**
ibm-6×*ibm-6* (n=24)	5.2±1.6**	3.1±2.4**	0.4±0.8$^{n.s}$
WT×*ibm1-6* (n=24)	5.0±1.0**	7.1±3.7**	0.3±0.6$^{n.s}$
ibm1-6×WT (n=22)	9.9±2.0**	16.7±7.1**	1.0±1.5*

Statistical analyses comparing WT×WT with each of *ibm-4*×*ibm-4,* WT×*ibm1-4, ibm1-4*×WT, *ibm-6*×*ibm-6,* WT×*ibm1-6* and *ibm1-6*×WT. * represents p-value<0.05, ** represents p-value<0.01, with two-tailed student *t* test.
