# Supplementary material for: Histone demethylase IBM1-mediated meiocyte gene expression ensures meiotic chromosome synapsis and recombination
Source: PLoS Genet. 2022 Feb 22;18(2):e1010041. doi: 10.1371/journal.pgen.1010041 (PMC8896719; doi:10.1371/journal.pgen.1010041)
Supplement: S2 Table — Statistical data were taken by comparing WT with ibm-4 or ibm1-6. * represents p-value<0.05, ** represents p-value<0.01, with two-tailed student t test. (PDF) [file pgen.1010041.s018.pdf]

S2 Table. Chiasmata frequency per chromosome in WT, *ibm1-4* and *ibm1-6*.

	Chr. 1	Chr. 2	Chr. 3	Chr. 4	Chr. 5
WT	2.53± 0.52	1.80± 0.41	2.27± 0.47	1.73± 0.46	2.33± 0.62
ibm1-4	1.79± 0.71**	1.33± 0.49**	2.00± 0.80$^{n.s}$	1.50± 0.60$^{n.s}$	1.85± 0.73*
ibm1-6	2.06± 0.85$^{n.s}$	1.28± 0.67*	2.05± 0.78$^{n.s}$	1.67± 0.62$^{n.s}$	2.00± 0.73$^{n.s}$

Statistical analyses comparing WT with *ibm-4* or *ibm1-6*. * represents p-value<0.05, ** represents p-value<0.01, with two-tailed student *t* test.
