# Supplementary material for: Histone demethylase IBM1-mediated meiocyte gene expression ensures meiotic chromosome synapsis and recombination
Source: PLoS Genet. 2022 Feb 22;18(2):e1010041. doi: 10.1371/journal.pgen.1010041 (PMC8896719; doi:10.1371/journal.pgen.1010041)
Supplement: S3 Table — (PDF) [file pgen.1010041.s019.pdf]

S3 Table. Expression of different meiotic genes in different genotype.

Gene ID	Gene name	ibm1-6-meiocyte-fold change	p-value	ibm1-6-leaf--fold change	p-value	ibm1-6 cmt3-meiocyte-fold change	p-value
AT5G51330	SWI1	4.770354751	7.10711E-69	-0.256343921	0.749514	4.126286246	1.454E-51
AT1G67370	ASY1	3.701483228	2.37931E-57	2.24479615	0.000483	2.609551243	4.201E-37
AT4G11080	3xHMG	3.691779813	8.07523E-58	0.35696939	0.055249	1.534347376	4.953E-11
AT5G05490	SYN1	3.657202767	2.64412E-42	0.504554997	0.619874	2.983603799	4.712E-28
AT1G14750	SDS	3.541388752	1.5298E-55	0.911469473	0.085403	2.644378421	2.054E-39
AT1G01690	ATPRD3	3.32619809	1.0715E-40	0.781484459	0.00501	2.832719828	9.05E-32
AT3G22880	ATDMC1	3.299135574	6.93211E-66	0.441283784	0.047451	2.409999589	1.346E-37
AT1G60930	RECQ4	3.292010387	9.14486E-50	-0.107576786	0.721661	2.206640813	4.868E-21
AT1G10710	PHS1	3.287014238	6.87031E-43	1.481888149	0.077861	2.293168606	1.115E-20
AT3G27730	RCK	3.186322537	3.88055E-57	1.152188689	0.018273	2.329744712	9.6E-29
AT5G52290	SHOC1	3.1683708	1.78087E-31	-0.142480954	0.843219	3.062624481	4.585E-27
AT2G46980	ASY3	3.015004966	1.06384E-52	0.481922258	0.037711	2.31711145	6.936E-27
AT4G17380	MSH4	2.833156795	5.7323E-31	0.657437691	0.401623	2.20278528	1.427E-23
AT3G06030	ANP3	2.808394964	1.30298E-34	1.066798993	5.41E-10	1.651655589	4.227E-10
AT2G21800	ATEME1A	2.782319618	6.47117E-12	0.125449213	0.778677	2.132028917	8.239E-06
AT5G57450	XRCC3	2.770244811	4.88996E-12	0.85091615	0.147997	2.474592862	1.486E-07
AT1G53490	HEI10	2.719585518	2.31927E-26	0.684375776	0.122468	1.378481335	1.493E-08
AT1G12790	PTD	2.718721156	9.46565E-43	0.377303629	0.319816	2.060153172	9.609E-26
AT5G21150	AGO9	2.711330657	1.19093E-26	-0.461025455	0.535614	1.848651026	3.766E-17
AT5G48390	ATZIP4	2.572703147	2.4615E-20	0.083606492	0.931149	2.356295452	6.334E-30
AT3G10440	SGO1	2.556221145	2.51568E-27	0.301155876	0.215122	1.13561018	8.33E-05
AT5G48600	SMC3	2.555030159	3.32046E-27	-0.048298809	0.756268	2.157616922	1.321E-20
AT1G22275	ZYP1b	2.502167089	3.78279E-27	-0.356762004	0.452061	1.72074815	3.289E-18
AT5G01630	BRCA2B	2.40521395	6.84847E-23	0.072506874	0.748466	2.239039688	1.144E-21
AT2G45280	ATRAD51C	2.404150816	2.73513E-30	0.947099738	0.000632	1.169773279	2.37E-07
AT3G27120	FIGL1	2.377516468	4.59497E-19	-0.011391439	0.967775	1.930801021	5.69E-13
AT1G04650	FLIP	2.372886782	1.99124E-19	0.037262315	0.911678	1.958031631	1.052E-14
AT4G15890	CAP-D3	2.367049454	4.11143E-23	0.016463858	0.954593	1.831325796	1.591E-11
AT4G14180	AtPRD1	2.322428385	3.46674E-21	-0.242832489	0.711863	1.639669067	4.853E-10
AT4G22970	AESP	2.268303011	2.25927E-20	0.052132987	0.816761	1.963124338	1.044E-12
AT4G29170	ATMND1	2.228807906	7.76973E-21	0.924821719	0.004096	2.078805369	3.15E-24
AT1G34355	ATPS1	2.217891315	1.07203E-26	0.767235669	0.000411	1.941023758	3.433E-14
AT1G22260	ZYP1a	2.135906132	9.17462E-21	0.727255589	0.162217	1.773440736	8.762E-20
AT3G13170	ATSPO11-1	2.133146151	4.37563E-14	-0.776117711	0.444825	1.84983502	2.717E-09
AT3G52115	ATCOM1	2.118028204	1.19753E-12	0.032505125	0.926073	1.720362415	1.729E-06
AT5G15540	SCC2	2.090847961	5.29878E-24	-0.107271483	0.43984	1.905690004	2.501E-22
AT5G62410	ATCAP-E1	2.064542292	2.8445E-20	0.106983837	0.477776	1.462869523	3.98E-09
AT3G15150	MMS21	2.035992791	3.43999E-14	-0.261332289	0.458854	1.785542816	6.4E-09
AT4G14970	FANCD2	2.027031578	1.14223E-18	-0.911924468	0.002022	1.848117875	8.613E-16
AT2G27170	SMC3	2.02325099	5.9799E-21	0.007031129	0.956645	1.884777425	2.932E-18
AT3G20475	ATMSH5	2.007533232	1.1187E-19	-0.824564763	0.114159	1.649031966	4.583E-18
AT1G01370	HTR12	2.003057671	5.77739E-13	0.493424207	0.121486	1.024206797	0.0059008
AT3G25980	MAD2	1.986136947	2.47116E-15	0.688797986	0.001433	0.250590237	0.5075412
AT2G31970	RAD50	1.981329248	1.07124E-17	0.022102596	0.888542	2.058181264	4.457E-19

S3 Table. Expression of different meiotic genes in different genotype (Extended).

Gene ID	Gene name	ibm1-6-meiocyte-fold change	p-value	ibm1-6-leaf--fold change	p-value	ibm1-6 cmt3-meiocyte-fold change	p-value
AT2G47980	SCC3	1.976600809	1.97361E-22	-0.041739579	0.742688	1.86508443	3.178E-20
AT4G24710	TRIP13	1.958989881	8.15855E-11	-0.080359582	0.815894	1.469500444	4.638E-05
AT4G21270	ATK1	1.955299189	1.43766E-09	0.439477589	0.032389	1.411592471	8.557E-06
AT5G07660	SMC6A	1.939852447	3.59144E-10	0.473935267	0.124381	1.605415226	6.003E-05
AT4G00020	BRCA2A	1.938872881	1.31928E-11	0.099985546	0.55568	2.56224177	9.226E-21
AT1G60460	TOPVIB	1.928481193	1.21066E-11	0.818453747	0.128839	0.172134221	0.6476706
AT1G77600	PDS5B	1.926564169	2.87339E-16	0.209991213	0.239911	1.894973809	6.793E-16
AT3G43210	TES	1.89693082	4.71283E-20	0.010636861	0.961195	1.043319756	4.935E-07
AT5G54260	MRE11	1.878159044	2.80181E-15	-0.242801296	0.321614	1.363272585	2.809E-07
AT1G03180	COMET	1.874633523	4.80684E-11	0.218934267	0.614047	1.804691116	7.117E-11
AT5G63920	TOP3A	1.858476451	1.62815E-13	0.257531823	0.287717	1.246543668	6.696E-06
AT1G13330	AHP2	1.852900341	1.03E-09	0.725696179	0.097971	1.95480275	1.317E-10
AT1G63990	SPO11-2	1.789490273	3.50166E-14	-1.050462727	1.56E-05	1.76555257	1.245E-13
AT5G04320	SGO2	1.768893571	2.95343E-10	0.340022933	0.073802	1.106185478	2.578E-05
AT1G06660	JASON	1.756235939	5.96759E-13	0.082146375	0.768087	1.485848494	1.176E-13
AT5G61460	SMC6B	1.66930873	7.55186E-05	0.056118753	0.802128	1.414693665	0.0005062
AT1G77390	TAM	1.599319741	6.27942E-14	0.854738545	0.042373	1.041859363	5.187E-06
AT1G35530	FANCM	1.592904318	5.23966E-07	0.352138786	0.242084	1.099461214	0.004948
AT2G06510	RPA1A	1.566118086	1.28506E-08	0.548027223	0.016908	1.722996061	1.493E-12
AT3G02980	MCC1	1.5511328	0.509315724	-0.15012279	0.819232	-2.004396399	0.6785443
AT5G57880	MPS1	1.543992522	1.12308E-10	0.734579011	0.000483	0.375229296	0.2214888
AT5G64520	ATXRCC2	1.513872547	2.56131E-08	-0.542439034	0.109125	0.616174336	0.0926026
AT3G48750	CDKA1	1.492561844	2.50819E-08	-0.007666419	0.954007	1.489830314	4.587E-10
AT3G47460	ATSMC2	1.43012981	2.04392E-07	-0.61808072	0.000529	0.503978672	0.1564353
AT1G78790	MHF2	1.423374459	2.05553E-07	-0.029289986	0.905596	1.097704644	0.00049
AT4G35520	MLH3	1.399632175	1.03507E-06	0.849262728	0.119463	0.590974902	0.098102
AT3G09660	MCM8	1.351476608	0.000100994	0.023155562	0.94962	1.682367332	4.5E-06
AT3G57860	GIG1	1.332468355	2.31366E-09	0.507715484	0.132122	0.631862346	0.0033709
AT5G40820	ATR	1.296732242	6.33805E-06	-0.176148385	0.529393	1.522837496	1.35E-07
AT3G54670	SMC1	1.266643158	8.19171E-09	0.179515366	0.213178	1.158457765	4.312E-08
AT5G48720	XRI	1.257698328	3.4422E-07	0.410602106	0.073867	1.353421762	1.436E-08
AT1G10930	ATSGS1	1.245308629	7.85027E-06	0.328236007	0.032362	1.631025883	2.821E-09
AT1G07745	ATRAD51D	1.206392356	0.000125971	0.358477891	0.212521	0.603389079	0.1204887
AT5G50930	MHF1	1.166796067	0.000405989	0.21232161	0.657109	0.564412854	0.1003518
AT5G03470	PP2AB'a	1.141617223	0.000506089	-0.246841291	0.149249	1.037392517	0.002135
AT4G20900	MS5	1.103577074	3.13222E-06	-0.628534162	0.716765	0.628574424	0.0053459
AT5G15920	SMC5	1.084502311	0.001048605	-0.092805079	0.597851	1.040751948	0.0081701
AT4G05190	ATK5	1.051290293	0.00017431	0.598432107	0.000524	0.263167014	0.4707681
AT3G59550	SYN3	1.03224293	0.001282383	1.021412034	0.000118	1.4953176	5.775E-06
AT4G18470	SNI1	1.029820146	0.014553947	0.485113951	0.291847	1.323125289	0.0010178
AT5G63540	ATRMI1	1.027704635	0.006717105	-0.627335853	0.18303	1.380050023	0.0015541
AT1G80810	PDS5D	1.01837832	0.000115408	0.179184429	0.362915	0.711929725	0.0020996
AT5G45400	RPA70C	0.99867596	0.00067229	0.139922288	0.610786	0.630538436	0.1432565
AT3G02680	NBS1	0.964158924	0.011005937	0.780523045	0.00934	1.693167439	5.304E-06

S3 Table. Expression of different meiotic genes in different genotype (Extended).

Gene ID	Gene name	ibm1-6-meiocyte-fold change	p-value	ibm1-6-leaf--fold change	p-value	ibm1-6 cmt3-meiocyte-fold change	p-value
AT5G40840	SYN2	0.938544814	0.004891363	0.556981602	0.123175	0.547846195	0.1940813
AT4G31880	PDS5C	0.920078012	9.37051E-05	-0.057822235	0.615054	0.97392382	6.531E-06
AT4G31400	CTF7	0.901134903	0.004476212	0.511349812	0.141001	0.892036164	0.0202364
AT3G33520	ARP6	0.867029716	0.000766265	0.925698653	2.04E-10	0.621198433	0.0413298
AT4G02460	PMS1	0.776491867	0.013689695	-0.193195772	0.423846	0.460519758	0.2383879
AT3G09880	PP2AB'b	0.759094664	0.011091992	0.119853742	0.43403	0.775225361	0.0067217
AT4G09140	ATMLH1	0.735506088	0.015921008	-0.649322506	0.004082	0.841155312	0.0028183
AT3G48190	ATM	0.719041032	0.006011454	-0.009371465	0.944957	1.191336855	9.909E-08
AT5G22010	AtRFC1	0.687517066	0.001275912	-0.179061589	0.240377	1.011868	6.221E-06
AT1G51130	NSE4A	0.686150047	0.02238871	0.348858811	0.273424	0.627925005	0.0674184
AT3G18524	MSH2	0.57116357	0.089311975	0.150907554	0.443505	0.815389027	0.031626
AT1G05180	AXR1	0.551616642	0.030552306	0.310780203	0.039836	0.766677349	0.0035248
AT5G20850	ATRAD51	0.492296703	0.201024546	-0.147578078	0.686634	0.470547441	0.1888162
AT3G25100	CDC45	0.42908957	0.293019925	-0.696362539	0.023683	0.58178614	0.2145976
AT4G01370	ATMPK4	0.398782202	0.159232462	0.066308189	0.594703	-0.246741873	0.435984
AT1G10970	ZIP4	0.381883497	0.222225378	-0.896504719	0.00028	-0.017480533	0.9543651
AT3G14190	PANS1	0.270981423	0.39553862	1.026173326	4.22E-05	0.110240232	0.6773522
AT5G63370	CDKG1	0.243196919	0.292146619	-0.121759806	0.283375	-0.632118307	0.0042172
AT1G66170	MMD1	0.226713931	0.395513368	-0.010259218	0.995517	-0.484510041	0.0701765
AT1G11060	WAPL1	0.014801947	0.959254825	-0.064755534	0.734002	-0.16326064	0.7084249
AT5G19400	SMG7	0.012409371	0.954295803	0.022058049	0.839745	0.295366636	0.1442198
AT1G27720	TAF4B	-0.192559336	0.553821177	-0.15659824	0.823796	0.023560229	0.9211444
AT2G42890	ML2	-0.233635152	0.618712652	-0.212694972	0.213556	0.090899702	0.835825
AT1G15110	PSS1	-0.249649378	0.516403646	0.134532078	0.628106	-0.011542288	0.9777975
AT1G08620	JMJ16	-0.257318377	0.38554038	-0.037487546	0.806042	0.536645267	0.0784612
AT1G75950	ASK1	-0.310579216	0.137924488	-0.213341362	0.060506	-0.62294511	0.0002648
AT4G33270	CDC20.1	-0.314114518	0.136802742	1.128501983	3.83E-19	-0.552414683	0.009959
AT5G61960	ML1	-0.380207121	0.214081613	-0.201142843	0.069988	-0.025476555	0.9207814
AT1G15940	PDS5E	-0.597383222	0.01199478	-0.065972321	0.713642	0.144536532	0.5076138
AT2G28130	ASAP1	-1.117795365	0.020973839	-0.184004726	0.498544	-0.941942589	0.1176617
AT5G47690	PDS5A	-1.140828467	4.0907E-07	-0.204470568	0.060459	0.110308224	0.5789099
AT1G29400	ML5	-1.518735642	4.22639E-05	-0.121690216	0.261832	-0.20756004	0.5198753
AT4G18120	ML3	-1.564556338	8.24341E-07	-0.207140061	0.171515	-0.958539379	0.0002237
AT4G30870	ATMUS81	-1.595980894	1.70866E-05	-0.195151095	0.599543	-0.37576108	0.3023751
AT5G16270	SYN4	-2.095198177	6.19823E-13	-0.244799117	0.034896	-1.315105746	4.356E-05
AT3G20760	NSE4B	-2.292096329	0.110443376	-10.70052419	5.91E-13	1.127315295	0.1373636
AT1G61030	WAPL2	-2.362830552	3.08357E-17	-0.345213185	0.079453	0.567559173	0.0116843
AT5G07290	AML4	-2.386914286	6.88465E-09	-0.372098602	0.007713	-1.552160776	9.887E-06
AT5G42190	ASK2	-2.44831369	1.5308E-09	0.3040251	0.009382	-1.878608013	6.463E-12
