# Supplementary material for: Histone demethylase IBM1-mediated meiocyte gene expression ensures meiotic chromosome synapsis and recombination
Source: PLoS Genet. 2022 Feb 22;18(2):e1010041. doi: 10.1371/journal.pgen.1010041 (PMC8896719; doi:10.1371/journal.pgen.1010041)
Supplement: S4 Table — Five siliques were analyzed in each plant. Statistical data were taken by comparing each AML5/ibm1-6 plant with ibm-6. Number of plants (#1-#2) were ranked by normal seed numbers. n.s represents not significant, * represents p-value<0.05, ** represents p-value<0.01, *** represents p-value<0.001 with two-tailed student t test. (PDF) [file pgen.1010041.s020.pdf]

S4 Table. Fertility analysis of 20 *AML5*/*ibm1-6* plants.

	Silique length (mm)	Normal seeds number	Aborted seeds number
WT	14.8±0.6	49.2±4.6	0
ibm1-6	5.6±0.4	5.8±1.6	0.4±0.6
# 1	5.7±0.4$^{n.s}$	4.4±1.3$^{n.s}$	0.4±0.5$^{n.s}$
# 2	5.5±0.4$^{n.s}$	4.6±1.7$^{n.s}$	0.8±0.8$^{n.s}$
# 3	5.4±0.8$^{n.s}$	4.8±1.5$^{n.s}$	0.4±0.5$^{n.s}$
# 4	5.8±0.6$^{n.s}$	5.0±1.6$^{n.s}$	1.2±1.3$^{n.s}$
# 5	5.6±0.4$^{n.s}$	5.2±1.3$^{n.s}$	0.6±0.5$^{n.s}$
# 6	6.2±1.0$^{n.s}$	6.4±3.4$^{n.s}$	1.0±1.0$^{n.s}$
# 7	6.1±0.4$^{n.s}$	7.2±0.8$^{n.s}$	1.0±0.7$^{n.s}$
# 8	6.0±0.4$^{n.s}$	7.4±0.9$^{n.s}$	0.6±0.5$^{n.s}$
# 9	6.6±0.7*	8.2±0.8*	0.6±0.9$^{n.s}$
# 10	7.0±0.8**	9.2±1.6*	0.4±0.5$^{n.s}$
# 11	7.6±0.7***	9.8±1.9**	0.6±0.5$^{n.s}$
# 12	8.9±1.1***	11.8±4.4*	1.6±0.5**
# 13	9.5±0.5***	13.4±2.9***	1.4±1.1$^{n.s}$
# 14	9.3±0.8***	14.0±3.5**	1.2±1.3$^{n.s}$
# 15	9.8±0.6***	14.6±4.3**	0.8±0.8$^{n.s}$
# 16	9.9±0.9***	15.4±4.9**	0.6±0.9$^{n.s}$
# 17	9.8±1.0***	15.6±2.8***	1.8±0.8$^{n.s}$
# 18	9.7±0.4***	16.0±3.5***	1.4±1.1$^{n.s}$
# 19	9.1±0.4***	16.8±3.7***	0.8±1.3$^{n.s}$
# 20	10.8±1.0***	18.2±2.1***	1.4±0.9$^{n.s}$

Five siliques were analyzed in each plant. Statistical analyses comparing each *AML5*/*ibm1-6 plant* with *ibm-6*. Number of plants (#1-#20) were ranked by normal seed numbers. n.s represents not significant, * represents p-value<0.05, ** represents p-value<0.01, *** represents p-value<0.001 with two-tailed student *t* test.
