# Supplementary material for: Histone demethylase IBM1-mediated meiocyte gene expression ensures meiotic chromosome synapsis and recombination
Source: PLoS Genet. 2022 Feb 22;18(2):e1010041. doi: 10.1371/journal.pgen.1010041 (PMC8896719; doi:10.1371/journal.pgen.1010041)
Supplement: S5 Table — (PDF) [file pgen.1010041.s021.pdf]

S5 Table. Primers used in this study.

Purpose	Primer	Sequence (5'-3')
**Genotyping**	ibm1-6-LP	TACCTGCAACCATTACAAGCC
	ibm1-6-RP	CTCTGGCATGTAAGGAGGATG
	ibm1-4-LP	GCTGCTACCACTAGTTGCCAG
	ibm1-4-RP	ACTGCCACGATAATGAGGTTG
	aml1-LP	ACCGTATATTACGTGGGGAGG
	aml1-RP	TCTTCTCGGTTTTCAGAAACG
	aml3-LP	CCAGAGGCAATAACGTGATTC
	aml3-RP	TCAAAGGAACTTGCGTGAAAC
	aml4-LP	AGGTGACCCAACAGAATTGTG
	aml4-RP	TCCGAAATTATGGCAAGACAC
	aml5-LP	GCTCAAAACAAAAAGCTCACG
	aml5-RP	TGCAAGCAAGAAACCATAACC
	suvh4-LP	CGGGAAAGAAAGAGGACAAAC
	suvh4-RP	ACTACCAACCAACCTGGAAGG
**Construction**	ProIBM1-F1	CGGAATTCGAGAATCCGCGTGATTTGTTTG
	ProIBM1-R1	CGAGCTCCCGTTCTCTTCTTCAACCCTAAC
	1306-IBM1-F1	CGGGATCCATGGATTCTGTGGAGGAAGAAG
	1306-IBM1-R1	GCGTCGACCATCTTCTCCATTTCTAATCTG
	ACT7-ML5-F1	TTTTTTTAGTGAAAAGGATCCATGGATATTCCACATGAAGCAGAA
	ACT7-ML5-R1	TCCAAGGGCGAATTGGTCGACGCTTCTCTCTCTGCCATCATC
**Real-time**	IBM1-real-F1	TGGCATGTAAGGAGGATGTG
	IBM1-real-R1	TCTAGCTTATTCGCCGGCTTT
	IBM1-real-F2	GTGCTGAAACGACTACTTCCG
	IBM1-real-R2	CATCGCTCCAAAACCGTTTC
	IBM1-real-F3	CCAGCATGGATCACTTAAGG
	IBM1-real-R3	GATCATATCGGCCATCCGTG
	IBM1-real-F4	AGCTCCACTGCGACATGTCTG
	IBM1-real-R4	GAGATCTTGTTCGGCATGCTT
	AML3-real-F	TGGCTTATGCACGAATCCAAGG
	AML3-real-R	GCTGCGAATTCTCCCGGATG
	AML4-real-F	CGTGGAGTGGGAGTAGATGG
	AML4-real-R	CCACATCATGCCACTTGACTG
	AML5-real-F	CTGCTCGAATGGCTATGAGG
	AML5-real-R	GGTTAAACACCACTAATGTCCC
**Chop PCR**	ML3-GB-F	CTGTGGTCCGGTTAGTTTAC
	ML3-GB-R	CGTGCATAAGCCAATGAAGC
	ML4-GB-F	AAGGATCCCATCGTGGTAAC
	ML4-GB-R	TGCTACCGGGAGAACCTAAG
	ML5-GB-F	AGACGATACGGCTGTTTCTC
	ML5-GB-R	GCTGTGAGTTCAGAGTCTTC
	DCL3-GB-F	TTTGGCACCCTGCCTTTATG
	DCL3-GB-R	ATGAGGCATCCACCTCAAAG
